# CD71-Mediated Effects of Soluble Vasorin on Tumor Progression, Angiogenesis and Immunosuppression

**DOI:** 10.3390/ijms26104913

**Published:** 2025-05-20

**Authors:** Yuechao Zhao, Can Xiao, Shaohua Li, Aixue Huang, Hui Li, Jie Dong, Qiaoping Qu, Xuemei Liu, Bo Gao, Ningsheng Shao

**Affiliations:** Department of Biochemistry and Molecular Biology, Beijing Institute of Basic Medical Sciences, Beijing 100850, China; zhaoyuechao@bmi.ac.cn (Y.Z.); xiaocan@bmi.ac.cn (C.X.); shhli55@163.com (S.L.); huangaixue@bmi.ac.cn (A.H.); lihui1@bmi.ac.cn (H.L.); dongjie@bmi.ac.cn (J.D.); qvqiaoping@163.com (Q.Q.); liuxuemei@bmi.ac.cn (X.L.)

**Keywords:** sVASN, CD71, tumor progression, angiogenesis, immunosuppression

## Abstract

Increasing recognition of the importance of the tumor microenvironment (TME) in cancer therapeutic strategies has led to more efforts to target molecules in the TME. Vasorin (VASN) is a transmembrane glycoprotein that can be cleaved and released into the extracellular matrix in a soluble form (sVASN), which is regarded as a decoy that inhibits the TGF-β signaling pathway. VASN is upregulated under hypoxic or tumorigenic conditions to regulate tumor progression. In this study, cell surface CD71 was identified as a specific binding protein of sVASN and mediated the internalization of sVASN in cancerous, endothelial and T cells. Endocytosed sVASN enhanced the nuclear translocation of p-STAT3(Tyr705), leading to the activation of a cascade of genes, ultimately contributing to tumor malignant progression. In cancer cells, sVASN promoted cell proliferation and migration by upregulating the YAP1/TAZ or mTOR-AKT pathways and it promotes stemness maintenance by regulating Notch1. In endothelial cells, sVASN facilitated angiogenesis through the VEGF signaling pathway. In T cells, sVASN inhibited the activation of T cells through AKT pathway. This study elucidated the mechanism by which sVASN acts as a tumor-promoting factor to accelerate tumor malignant progression through cell-surface CD71 and presented sVASN as a novel target for cancer therapy.

## 1. Introduction

Tumor progression is a complicated and delicate process that requires the involvement of a multitude of cancerous or non-cancerous cells, described as the tumor microenvironment (TME) [1]. In addition, the proteins produced by these cells present in the tumor also support malignant progression. Accordingly, novel therapeutic targets within the TME, especially specific ones that target the extracellular ligand-receptor interactions of the TME, remain extremely promising.

Vasorin (VASN), a type I transmembrane glycoprotein, is composed of 673 amino acids and is encoded by the VASN gene (also known as SLIT-like 2). VASN contains functional structures, including tandem leucine-rich (LRR) domains, epidermal growth factor (EGF)-like domains and fibronectin III (FN3) domains. VASN was found to be a TGF-β-binding protein for the first time in 2004 and functions as a key cell surface protein and secretory protein in response to arterial damage [2]. Malapeira et al. reported that disintegrin and metalloprotease 17 (ADAM17) are involved in the cleavage and processing of VASN on the cell membrane to produce soluble VASN (sVASN) [3]. In normal tissues, VASN is usually expressed on the surface of vascular smooth muscle cells in large arteries [4]. An increasing number of studies have shown that VASN is highly expressed in different tumor cells to regulate tumor progression [5,6,7,8,9]. Our previous study revealed that sVASN secreted by liver cancer cells can be transferred to human umbilical vein endothelial cells (HUVECs) via exosomes to promote the migration of endothelial cells. In addition, blocking membrane VASN with a VASN antibody did not cause significant changes in the cell phenotype, indicating that the effect of VASN on liver cancer cells might be attributed to sVASN [10,11]. We are faced with questions regarding how sVASN reinternalizes into effector cells regardless of exosomes, given the ability of internalized exogenous sVASN to regulate cell functions.

CD71, alternatively referred to as transferrin receptor protein 1 (TFR1 or TFRC), is a type II transmembrane glycoprotein. It is widely expressed across nearly all cell and tissue types and represents the most pivotal regulatory receptor in the cellular iron metabolism process, which is considered a marker of endocytic recycling [12,13]. The expression of CD71 is influenced by the cellular iron content, cell differentiation and inflammation [14]. Excess iron promotes cancer development and is a specific marker of cancer [15,16]. In addition to its main function in iron intake, CD71 can also bind to HFE (hereditary hemochromatosis protein) as well as HCV to mediate internalization and regulate cell proliferation [17,18]. CD71 has also been shown to be important for T-cell activation and is considered a marker of T-cell activation [19,20].

In this study, we found that sVASN could specifically bind to CD71, which mediated the internalization of sVASN. The internalized sVASN could facilitate the proliferation, migration and maintenance of the stemness of cancer cells; promote the angiogenesis of endothelial cells; and inhibit the activation of T cells via the surface of CD71. This study elucidated the mechanism by which sVASN enhanced the nuclear translocation of p-STAT3 (Tyr705), which further initiates cascades of gene expression in different types of cells to accelerate tumor malignant progression. Our study is expected to offer new insights into the development of valuable drug targets for cancer therapy.

## 2. Results

### 2.1. CD71 Was Identified as a sVASN-Binding Protein

**Figure 1:**
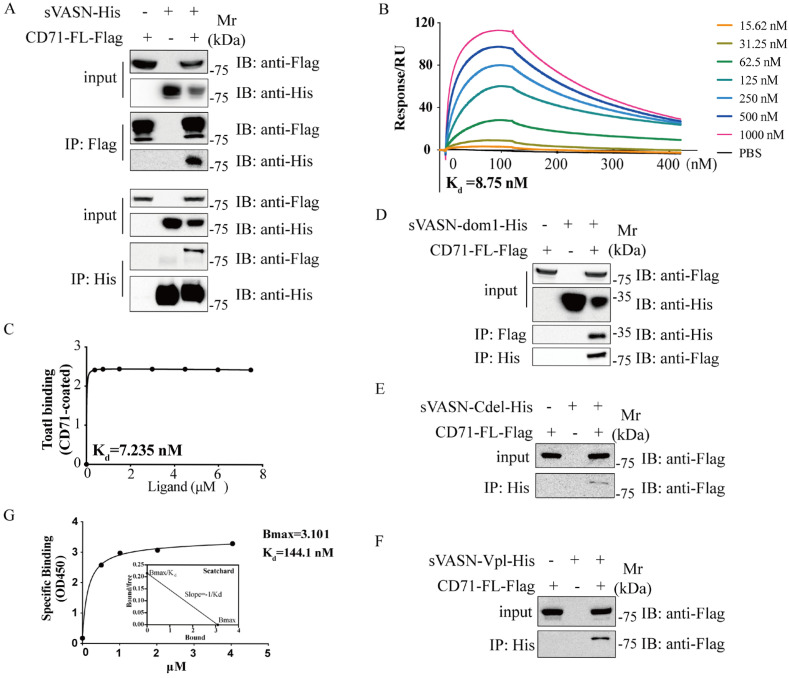
CD71 was identified as a sVASN-binding protein. (**A**) Co-IP analysis of the binding of sVASN to full-length CD71. The sVASN plasmid was constructed with a His tag, and the CD71 plasmid was constructed with a Flag tag, both of which are in the C-terminus. (**B**) BIAcore analysis of the binding of sVASN to CD71. CD71 was labeled with biotin at a concentration of 50 nM. (**C**) ELISA analysis of the binding of sVASN to CD71. CD71 was coated at a concentration of 10 µg/mL. The concentrations of sVASN used were 50, 40, 30, 20, 10, 5, 2.5 and 0 µg/mL. (**D**) Co-IP analysis of the binding of sVASN-dom1 to full-length CD71. sVASN-dom1, 1–240 aa of VASN with a His tag at the C-terminus. (**E**) Co-IP analysis of the binding of sVASN-Cdel to full-length CD71. sVASN-Cdel, the N-terminus of sVASN, is a 1–220 aa fragment of VASN with a His tag at the C-terminus. (**F**) Co-IP analysis of the binding of VASN-Vp1 to full-length CD71. sVASN-Vp1, 1–135 aa of VASN with a His tag at the C-terminus. (**G**) Saturation binding of sVASN to CD71. CD71 was coated at a concentration of 1 µg/mL. The binding substrate sVASN ranged from 200 µg/mL to 3.125 µg/mL. The equation for the one-site binding model: [Y = Bmax×X/(K_d_ + X)]. All of the assays were conducted in triplicate.

To identify the binding protein of sVASN, we previously adopted an immunoprecipitation (IP) assay combined with mass spectrometry (MS) to capture the binding proteins of sVASN in HepG2 hepatocellular carcinoma cell lysates and identified CD71 as a candidate binding protein (Supplementary Figure S1A). Further co-IP assays in HEK293T cells revealed that sVASN could bind to full-length CD71 (Figure 1A). The binding affinity was determined via BIAcore, and the association constant K_d_ of sVASN to CD71 was 8.75 nM (Figure 1B). An ELISA was also employed to determine the association constant K_d_ (Figure 1C and Supplementary Figure S1B). Interaction docking and molecular dynamics simulation analysis revealed that sVASN and CD71 (membrane-type) could form a stable complex on the cell surface and that the loop structure of sVASN formed “an arm” to lock CD71 (Supplementary Figure S1C,D). Considering the potential impact of the loop structure of sVASN on binding, we employed three truncated constructs: sVASN-dom1 (1–240 aa), sVASN-dom2 (240–380 aa) and sVASN-dom3 (380–575 aa). Only sVASN-dom1 was found to bind to CD71 (Figure 1D). We subsequently created two more groups of truncated constructs based on sVASN-dom1 and the 1–240 aa region of sVASN (Figure 1E,F and Supplementary Figure S1E–G). These results led us to conclude that the binding site of sVASN to CD71 is located at the N-terminal 1–85 aa of sVASN, which contains an LRR sequence (https://lrrsearch.com/index.php?page=tool, accessed on 20 August 2024), LQLLDLSQNQI (77–87 aa), the major role of which is to participate in protein–protein interactions and innate immunity [21]. Additionally, we analyzed the saturation binding range by adding an excess of sVASN (200:1) and proved that the association constant K_d_ of sVASN to CD71 was 144.1 nM and that the maximum receptor density (Bmax) was 3.101 (Figure 1G). Finally, we investigated the possible competitive binding between sVASN and Tf to CD71. According to a previous study and the PDB database (3S9N), there is no overlap between the binding sites of Tf to CD71 and those of sVASN to CD71. Moreover, we found that there was little competition between sVASN and transferrin (Tf) when sVASN was bound to CD71 (Supplementary Figure S1H).

### 2.2. sVASN Could Be Internalized into Different Types of Cells Through Cell Surface CD71

**Figure 2:**
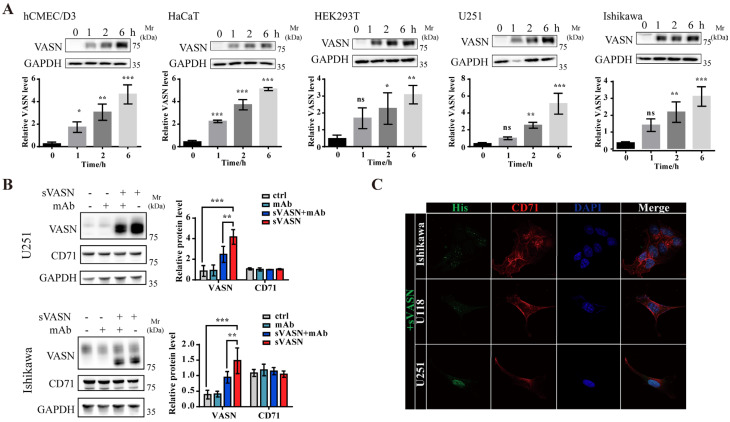
Exogenous sVASN could be internalized into different types of cells. (**A**) Western blot analysis of the internalization of exogenous sVASN into different types of cells. *n* = 3 (**B**) Western blot analysis of the internalization of exogenous sVASN into U251 or Ishikawa cells counteracted by a VASN-specific monoclonal antibody (mAb). *n =* 3 (**C**) Internalization of exogenous sVASN into different types of cells visualized under a laser scanning confocal microscope (LSCM). The Western blot bands were determined via ImageJ software (1.51j8). GAPDH was used as an internal control. All of the data are presented as the means ± SDs. All of the assays were conducted in triplicate. Statistical analysis in (**A**) was performed using ordinary one-way ANOVA followed by Dunnett’s multiple comparisons test and in (**B**) using two-way ANOVA followed by Sidak’s multiple comparisons test. * *p* < 0.05, ** *p* < 0.01, *** *p* < 0.001, ns not significant compared with the indicated groups.

The expression of VASN varied depending on the cell type (Supplementary Figure S2A). VASN and CD71 co-localized in untreated VASN high-expression cells on the cell membrane (Supplementary Figure S2B). However, whether the co-localized VASN was in a soluble form or a membrane form remains unclear. Western blot assays revealed that exogenous sVASN could be internalized into different types of cells in a time-dependent manner (Figure 2A). Next, we employed a specific anti-VASN ectodomain monoclonal antibody to counteract the addition of exogenous sVASN [22]. The results revealed that the mAb had little effect on the expression of CD71 but significantly inhibited the endocytosis of sVASN (Figure 2B). Confocal images also revealed that exogenous sVASN could be internalized into different types of cells (Figure 2C and Supplementary Figure S2C).

**Figure 3:**
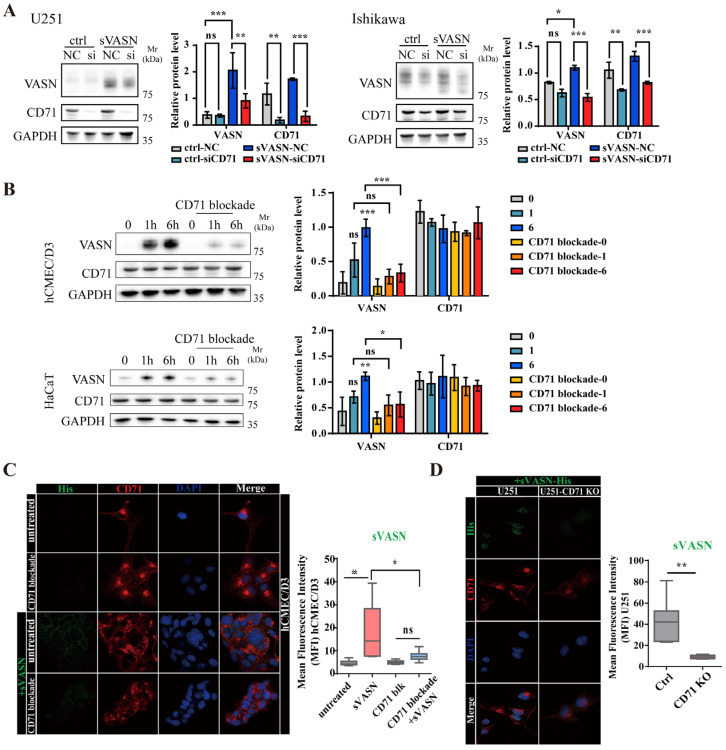
Exogenous sVASN could be internalized into different types of cells through cell surface CD71. (**A**) Western blot analysis of the internalization of exogenous sVASN into U251 or Ishikawa cells with siRNA treatment targeted to the CD71 gene. NC, negative control; si, siCD71; *n =* 3. (**B**) Western blot analysis of the internalization of exogenous sVASN in hCMEC/D3 or HaCaT cells with CD71 blockade treatment. *n =* 3. (**C**) Internalization of exogenous sVASN with CD71 blockade treatment visualized via LSCM. (**D**) Internalization of exogenous sVASN into the CD71 knockout cell line visualized via LSCM. The Western blot bands or the mean fluorescence intensity (MFI) were determined via ImageJ software. GAPDH was used as an internal control. All of the data are presented as the means ± SDs. All of the assays were conducted in triplicate. Statistical analysis in (**A**) was performed using two-way ANOVA followed by Sidak’s multiple comparisons test, in (**B**) using two-way ANOVA followed by Tukey’s multiple comparisons test, in (**C**) using ordinary one-way ANOVA followed by Tukey’s multiple comparisons test and in (**D**) using an unpaired *t* test. * *p* < 0.05, ** *p* < 0.01, *** *p* < 0.001, ns not significant compared with the indicated groups.

To assess whether CD71 mediates the endocytosis of sVASN into cells, the CD71 gene was silenced via siRNA, which resulted in a decrease in the VASN protein level (Figure 3A). To minimize disturbance of the CD71 gene, a specific blocking peptide of CD71 was used, which did not affect the expression of CD71 but significantly reduced the internalization of exogenous sVASN (Figure 3B). Exogenous sVASN could be internalized into different types of cells depending on the level of CD71 on the cell membrane, which was impeded after CD71 was blocked (Figure 3C and Supplementary Figure S3A). In addition, we employed Ferristatin II, an inhibitor of CD71 that could promote the degradation of CD71 both in vitro and in vivo. The inhibition of CD71 by Ferristatin II significantly reduced the internalization of exogenous sVASN (Supplementary Figure S3B). We also knocked out CD71 in U251 cells via CRISPR/Cas9 technology and found that the amount of sVASN internalized into CD71 knockout cells was significantly reduced (Figure 3D and Supplementary Figure S3C).

**Figure 4:**
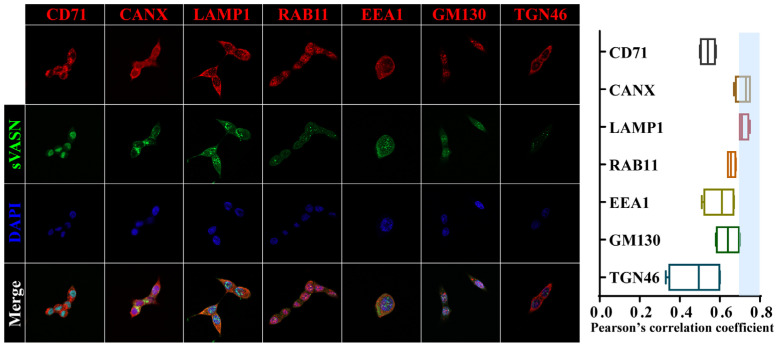
Co-localization of sVASN and different recycling endosomes in HCT116 cells visualized via LSCM. Endoplasmic reticulum (CANX), late endosome (LAMP1), recycling endosome (RAB11), early endosome (EEA1) and Golgi (GM130 and TGN46). Pearson correlation coefficient was determined via ImageJ software and assays were conducted in triplicate.

The endocytic recycling process of exogenous sVASN was determined in different types of cells, and the results revealed that sVASN was mostly co-localized with the endoplasmic reticulum (ER, CANX) and late endosomes (LAMP1) (Figure 4 and Supplementary Figure S4A,B). The endocytosis cycle of exogenous sVASN indicated that sVASN internalization into cells through CD71 might involve two pathways: one cycling to the ER for continued modification and processing and the other cycling to the late endosome for degradation, which might be an extensive pathway for exogenous proteins.

### 2.3. Endocytosed sVASN Enhanced the Nuclear Translocation of STAT3

**Figure 5:**
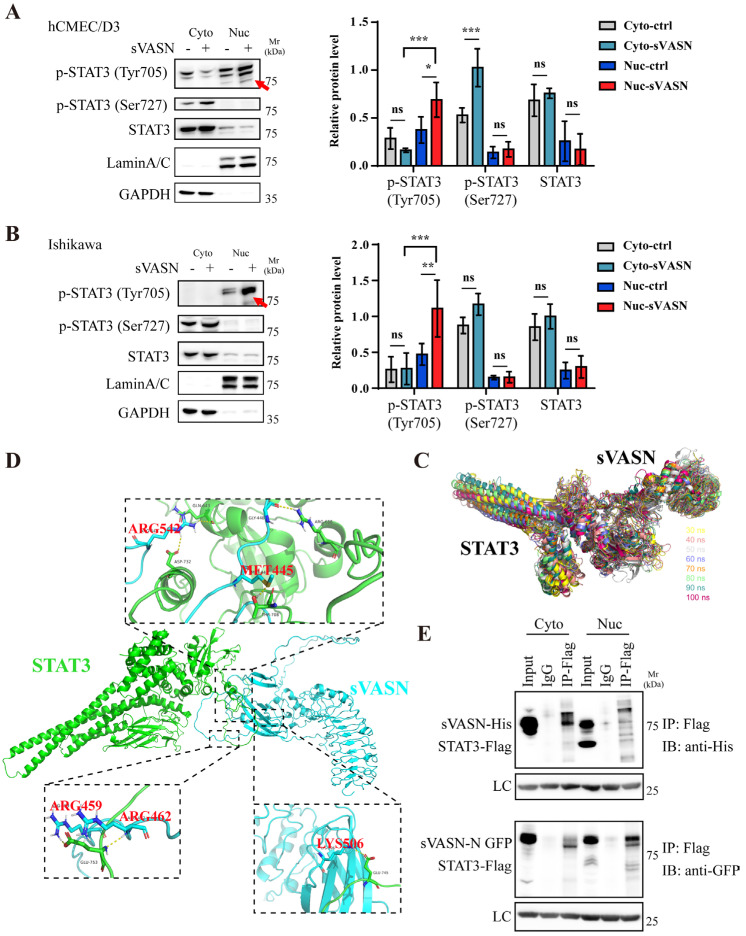
Endocytosed sVASN enhanced the nuclear translocation of STAT3. (**A**,**B**) Western blot analysis of the protein changes in STAT3 in the cytoplasmic or nuclear lysates of hCMEC/D3 (**A**) or Ishikawa cells (**B**), respectively. The red arrows indicate the p-STAT3 (Tyr705) levels with significant changes in the nucleus. (**C**) Simulation docking of the dynamic complex of sVASN-STAT3 at a subsequent time (30 ns–100 ns). *n =* 3. (**D**) Simulation docking of sVASN (green) with STAT3 (cyan). The interaction sites are marked in red. (**E**) Co-IP analysis of the binding of sVASN to STAT3 in cytoplasmic or nuclear lysates. sVASN, with a His or GFP tag in the N-terminus, and STAT3, with a Flag tag in the C-terminus. *n =* 3. The Western blot bands were determined via ImageJ software. GAPDH, LaminA/C or IgG-LC was used as an internal control in (**A**,**B**,**E**). All of the assays were conducted in triplicate. All of the data are presented as the means ± SDs. Statistical analyses in (**A**,**B**) were performed using two-way ANOVA followed by Tukey’s multiple comparisons test. * *p* < 0.05, ** *p* < 0.01, *** *p* < 0.001, ns not significant compared with the indicated groups.

As exogenous sVASN exhibited obvious nuclear localization (Figure 2C and Figure 4) and analysis of the sequence of VASN revealed that there was no nuclear localization signal (NLS), we speculated that the nuclear localization of sVASN might depend on other molecules. STAT3 is a vital transcription factor that promotes the rapid induction of gene expression involved in various basic cell functions to respond to cytokines, hypoxia, growth factors or stress [23,24,25,26,27]. Therefore, we investigated the possibility that sVASN shuttling from the cytosol to the nucleus is mediated by STAT3. We found that exogenous sVASN significantly increased both the levels of p-STAT3 (Tyr705) and p-STAT3 (Ser727), but only p-STAT3 (Tyr705) was related to nuclear translocation (Figure 5A,B). The interaction docking simulation revealed that sVASN and STAT3 gradually formed a stable complex conformation (Figure 5C,D). The co-IP results revealed that sVASN could bind to STAT3 directly in both the cytoplasm and the nucleus (Figure 5E).

**Figure 6:**
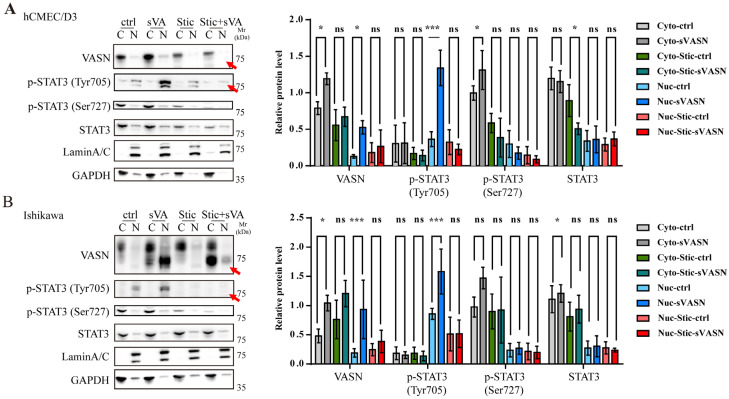
The nuclear translocation of sVASN was dependent on the phosphorylated STAT3 at tyrosine 705 site. (**A**,**B**) Western blot analysis of the protein changes in VASN and STAT3 in the cytoplasmic or nuclear lysates of hCMEC/D3 (**A**) and Ishikawa (**B**) cells, respectively, stimulated with exogenous sVASN combined with or without Stattic treatment. The red arrows indicate the protein levels with significant changes in the nucleus. *n =* 3. The Western blot bands were determined via ImageJ software. GAPDH or LaminA/C was used as an internal control. All of the assays were conducted in triplicate. All of the data are presented as the means ± SDs. Statistical analyses in (**A**,**B**) were performed using two-way ANOVA followed by Tukey’s multiple comparisons test. * *p* < 0.05, *** *p* < 0.001, ns not significant compared with the indicated groups.

Next, we employed Stattic, an inhibitor of STAT3 that selectively inhibits the nuclear translocation of STAT3 [28]. The level of p-STAT3 significantly decreased in both the nucleus and the cytoplasm after Stattic treatment. Intriguingly, VASN levels increased in the cytoplasm but declined in the nucleus, suggesting that sVASN was retained in the cytoplasm when STAT3 nuclear translocation was inhibited (Figure 6A,B). Collectively, our results also proved that the nuclear translocation of sVASN was related to the Tyr705 phosphorylation residue of STAT3 and that STAT3 also mediated the nuclear translocation of sVASN.

**Figure 7:**
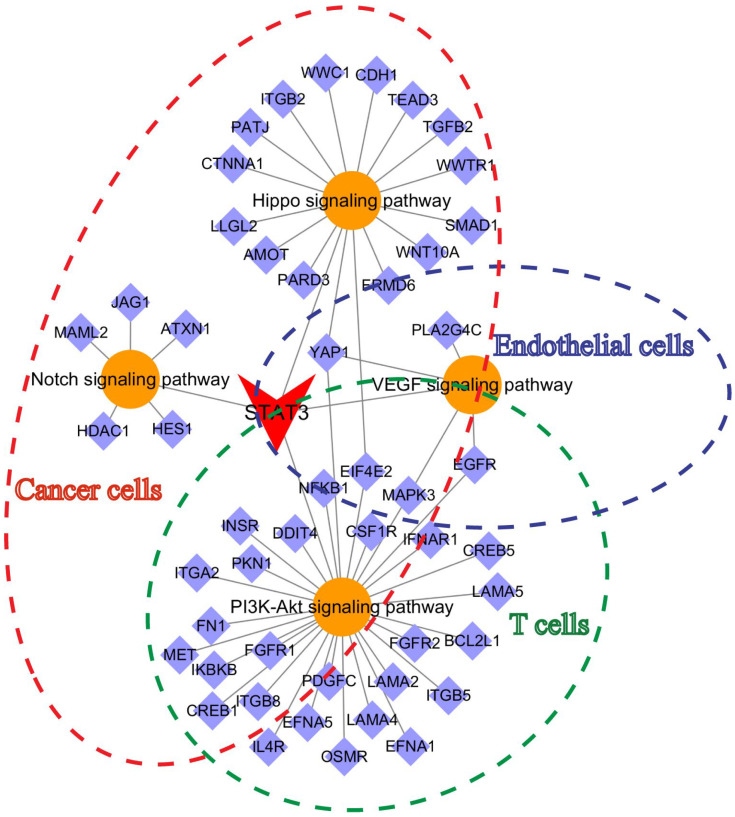
Summary of signaling pathways activated by exogenous sVASN treatment in different cell types. The red dashed line represents the signaling pathway activated by sVASN in cancer cells. The purple dashed line represents the signaling pathway activated by sVASN in endothelial cells. The green dashed line represents the signaling pathway activated by sVASN in T cells.

To obtain more evidence, the protein interaction network was predicted via the STRING and BioGrid databases to analyze the protein–protein interaction (PPI) networks of VASN. The results showed that VASN mainly interacted with STAT3 and TGF family members as reported (Supplementary Figure S5A). STAT3 signaling plays a crucial role in cancer cell proliferation and migration, as well as influencing angiogenesis, immunosuppression and cancer stem cell (CSC) self-renewal [29,30,31]. A previous study revealed that the phosphorylation of tyrosine 705 of STAT3 promotes STAT3 homodimerization and translocation to the nucleus, activating the transcription of target genes [32]. Thus, we generated STAT3-binding promoter tracks by using ChIP-seq datasets from the GEO database for the possible regulation of genes identified in PPI networks by STAT3. The distribution of reads obtained via ChIP-Seq was strongly associated with the Hippo and Notch signaling pathways in cancer cells or tumors, whereas the PI3K-AKT signaling pathway in primary human TH1 cells were shown as the robust tracks of the YAP1, Notch1 or AKT1 gene promoter region. (Supplementary Figure S5B). The above evidence led to the conclusion that sVASN enhanced the nuclear translocation of p-STAT3 (Tyr705). The increase in p-STAT3 (Tyr705) in the nucleus subsequently initiates downstream signaling pathways, including the Hippo and Notch pathways in cancer cells, the VEGF pathway in endothelial cells and the PI3K-AKT pathway in T cells (Figure 7).

### 2.4. Exogenous sVASN Was Conducive to the Proliferation, Migration and Stemness Maintenance of Cancer Cells Through Cell Surface CD71

**Figure 8:**
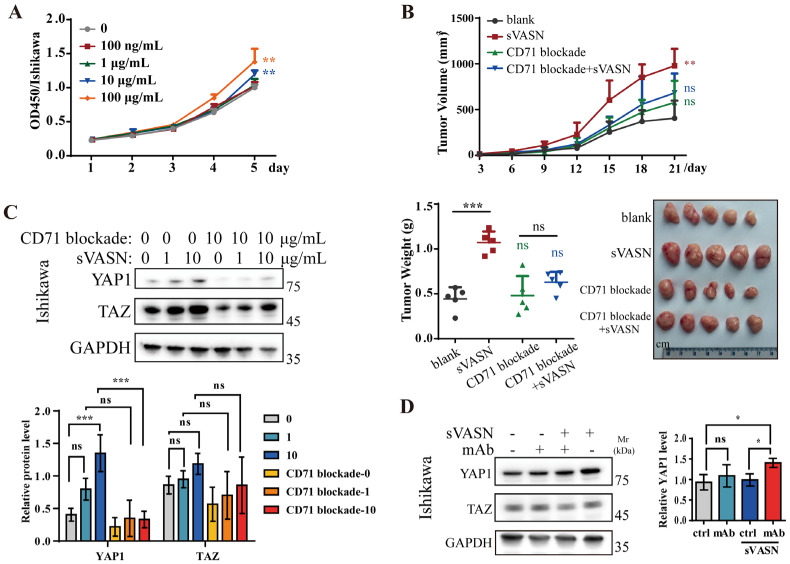
Exogenous sVASN was conducive to the proliferation of cancer cells through cell surface CD71. (**A**) Proliferation assay of Ishikawa cells treated with exogenous sVASN. (**B**) The tumorigenesis of Ishikawa cells in vivo after treatment with exogenous sVASN, CD71 blockade, CD71 blockade followed by sVASN or a blank. The tumor volumes (left panel) were measured every three days, and the tumor weights (middle panel) and images (right panel) of the tumors from the nude mice at autopsy are presented. *n =* 5. (**C**,**D**) Western blot analysis of the YAP1/TAZ signaling pathway in Ishikawa cells treated with sVASN with or without the CD71-blocking peptide (**C**), or with the counteracting VASN-specific monoclonal antibody (mAb) (**D**). *n =* 3. The Western blot bands were determined via ImageJ software. GAPDH was used as an internal control. All of the data are presented as the means ± SDs. All of the assays were conducted in triplicate. Statistical analyses in (**A**) and (**B**, left) were performed using one-way ANOVA with Dunnett’s multiple comparison test, in (**B**, right) and (**D**) using ordinary one-way ANOVA followed by Dunnett’s multiple comparisons test and in (**C**) using two-way ANOVA followed by Tukey’s multiple comparisons test. * *p* < 0.05, ** *p* < 0.01, *** *p* < 0.001, ns not significant compared with the indicated groups.

To clarify the function of sVASN, we performed biological studies with different types of cells. We found that the supernatant of HepG2 cells significantly promoted the proliferation of U118 and hCMEC/D3 cells but had little effect on U87 cells, whose constitutive expression of CD71 was extremely low (Supplementary Figure S6A,B). We next proved that exogenous sVASN promoted the proliferation of Ishikawa cells, especially at high concentrations (Figure 8A). To determine this effect in vivo, Ishikawa cells were pretreated with sVASN, followed by CD71 blockade. sVASN significantly increased tumor growth, including tumor size, volume and weight (Figure 8B) in vivo, and these effects were significantly attenuated after CD71 blockade. To verify our hypothesis, the signaling pathway was analyzed according to the ChIP-seq analysis. In Ishikawa cells, sVASN activates the YAP1/TAZ signaling pathway, manifesting as a gradual increase in the level of YAP1/TAZ proteins with increasing sVASN (Figure 8C). However, the signaling changes were hindered when the CD71-blocking peptide was employed, indicating the dominant characteristic of CD71. We employed the VASN monoclonal antibody to counteract the additional introduction to determine the specific role of sVASN. The results showed that the addition of mAb also attenuated the expression of YAP1/TAZ in Ishikawa cells (Figure 8D).

**Figure 9:**
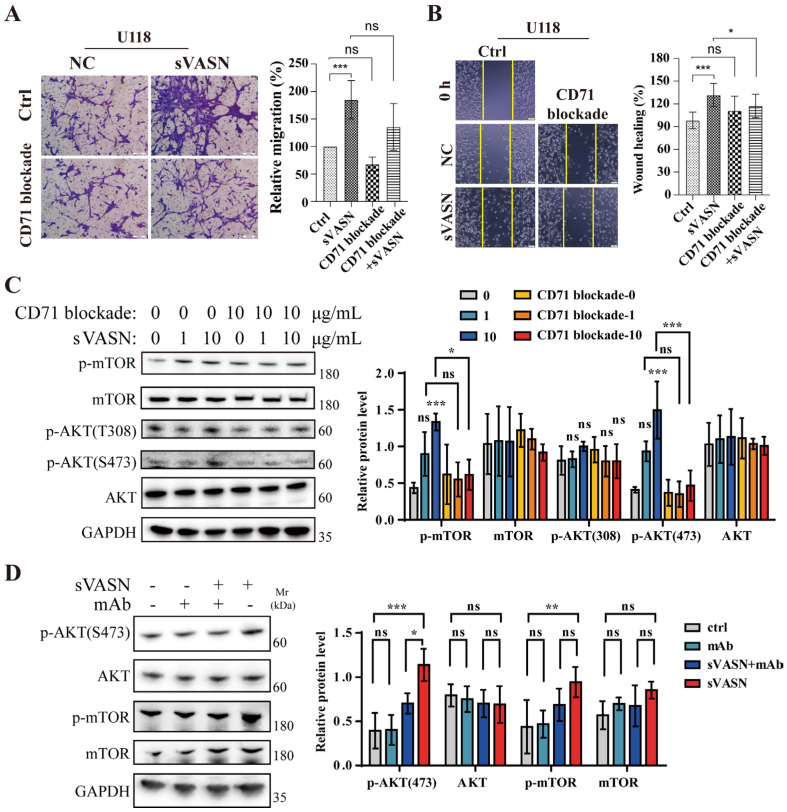
Exogenous sVASN was conducive to the migration of cancer cells through cell surface CD71. (**A**,**B**) Migration and wound healing assays of U118 cells treated with exogenous sVASN, CD71 blockade, CD71 blockade followed by sVASN or the blank control. Representative images (left) and statistics (right) are shown. (**C**,**D**) Western blot analysis of the mTOR-AKT signaling pathway in U118 cells treated with sVASN with or without CD71 blockade (**C**) or with VASN-specific monoclonal antibody (mAb) counteraction (**D**). *n =* 3. The Western blot bands were determined via ImageJ software. GAPDH was used as an internal control. All of the data are presented as the means ± SDs. All of the assays were conducted in triplicate. Statistical analyses in (**A**,**B**) were performed using ordinary one-way ANOVA followed by Tukey’s multiple comparisons test and in (**C**,**D**) using two-way ANOVA followed by Tukey’s multiple comparisons test. * *p* < 0.05, ** *p* < 0.01, *** *p* < 0.001, ns not significant compared with the indicated groups.

Additionally, exogenous sVASN significantly promoted cell migration, including migration and scratch healing, in U118 glioma cells but not in U87 cells (Figure 9A,B, Supplementary Figure S7A,B). However, the migratory-facilitating effect was significantly mitigated when CD71 was blocked. Examination of the signaling pathway revealed that sVASN activated the mTOR-AKT signaling pathway, especially the phosphorylation of AKT (S473) (Figure 9C). VASN monoclonal antibody counteraction assays demonstrated that sVASN promoted the migration of U118 cells through cell surface CD71 (Figure 9D). We also employed Ferristatin II to inhibit CD71 expression and found that cell migration was attenuated when CD71 was inhibited (Supplementary Figure S7C,D), indicating the endocytic role of CD71.

**Figure 10:**
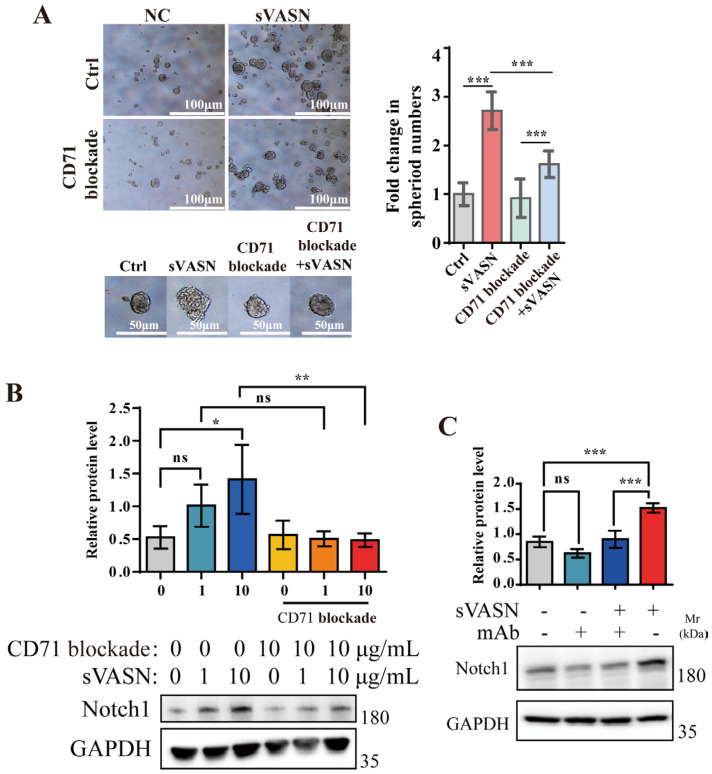
Exogenous sVASN was conducive to the stemness maintenance of cancer cells through cell surface CD71. (**A**) Spheroid formation abilities of exogenous sVASN, CD71 blockade, CD71 blockade followed by sVASN or the blank. Representative images of the number of spheroids (left), the size of the spheroids (bottom) and the statistics of the number (right) used in the assay are shown. Scale bar, 100 μm. *n =* 5. (**B**,**C**) Western blot analysis of the Notch signaling pathway in U251 cells with or without CD71-blocking peptide treatment (**B**) or VASN-specific monoclonal antibody (mAb) counteraction (**C**). *n =* 3. The Western blot bands were determined via ImageJ software. GAPDH was used as an internal control. All of the data are presented as the means ± SDs. All of the assays were conducted in triplicate. Statistical analyses in (**A**,**C**) were performed using ordinary one-way ANOVA followed by Tukey’s multiple comparisons test and in (**B**) using ordinary one-way ANOVA followed by Sidak’s multiple comparisons test. * *p* < 0.05, ** *p* < 0.01, *** *p* < 0.001, ns not significant compared with the indicated groups.

We next examined the spheroid formation abilities of U251 cells under exogenous sVASN treatment according to a previous study [33]. The results showed that sVASN could significantly promote the spheroid formation of U251 cells, including the size and number of spheroids (Figure 10A), the effect of which decreased after CD71 blockade. The signaling pathway analysis revealed changes in Notch1 expression in U251 cells (Figure 10B) which were reversed when the cells were treated with the blocking peptide (Figure 10B) or VASN monoclonal antibody (Figure 10C). Our results indicated that the ability of exogenous sVASN to promote the stemness of U251 cells was also dominated by CD71.

Finally, the immunofluorescence staining (IF) of VASN with CD71 or STAT3 was validated by using the bulk tumors shown in Figure 8B. VASN significantly co-localized with CD71 or STAT3, mainly in the sVASN treatment group (red) (Supplementary Figure S8A,B). In terms of the mean fluorescence density, the protein levels of VASN in the sVASN treatment group (red) were significantly greater than those in the other groups, indicating the persistent effect of sVASN on cancer proliferation (Supplementary Figure S8C).

### 2.5. sVASN Promoted the Angiogenesis of Endothelial Cells Through Cell Surface CD71

**Figure 11:**
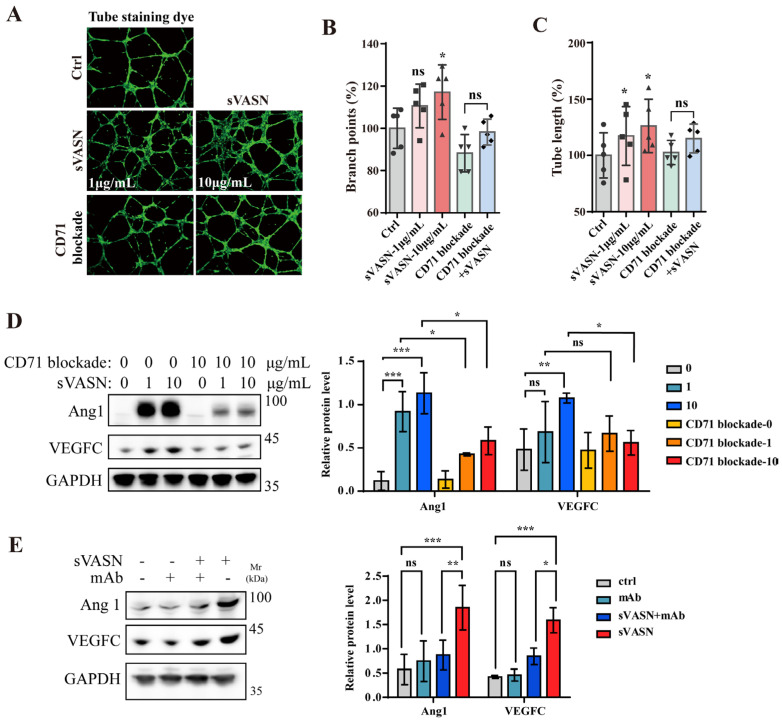
sVASN promoted the angiogenesis of endothelial cells through cell surface CD71. (**A**–**C**) Angiogenesis of brain microvascular endothelial cells (hCMECs/D3) treated with exogenous sVASN, CD71 blockade, CD71 blockade followed by sVASN or the blank. Representative images (**A**), statistics of branch points (**B**) and tube length (**C**) are shown. *n =* 6. (**D**) Western blot analysis of the VEGF signaling pathway in hCMEC/D3 cells treated with or without CD71 blockade. *n =* 3. (**E**) Western blot analysis of the VEGF signaling pathway in hCMEC/D3 cells treated with sVASN with or without a VASN-specific monoclonal antibody (mAb) counteraction. *n =* 3. The Western blot bands were determined via ImageJ software. GAPDH was used as an internal control. All of the data are presented as the means ± SDs. All of the assays were conducted in triplicate. Statistical analyses in (**B**,**C**) were performed using ordinary one-way ANOVA followed by Tukey’s multiple comparisons test and in (**D**,**E**) using two-way ANOVA followed by Tukey’s multiple comparisons test. * *p* < 0.05, ** *p* < 0.01, *** *p* < 0.001, ns not significant compared with the indicated groups.

In endothelial cells (hCMEC/D3), exogenous sVASN at different concentrations promoted angiogenesis (Figure 11A), including the branch points and tube length of new vessels (Figure 11B,C), which was significantly attenuated when CD71 was blocked, indicating the endocytic role of CD71 in endothelial cells. The signaling pathway analysis of hCMEC/D3 cells revealed that the key molecules of the VEGF signaling pathway related to angiogenesis, including VEGFC and Ang1, were significantly increased, as reported (Figure 11D) [34,35]. Monoclonal antibody counteraction assays demonstrated that sVASN promoted the angiogenesis of endothelial cells through cell surface CD71 (Figure 11E).

### 2.6. sVASN Inhibited T Cell Activation Through Cell Surface CD71

**Figure 12:**
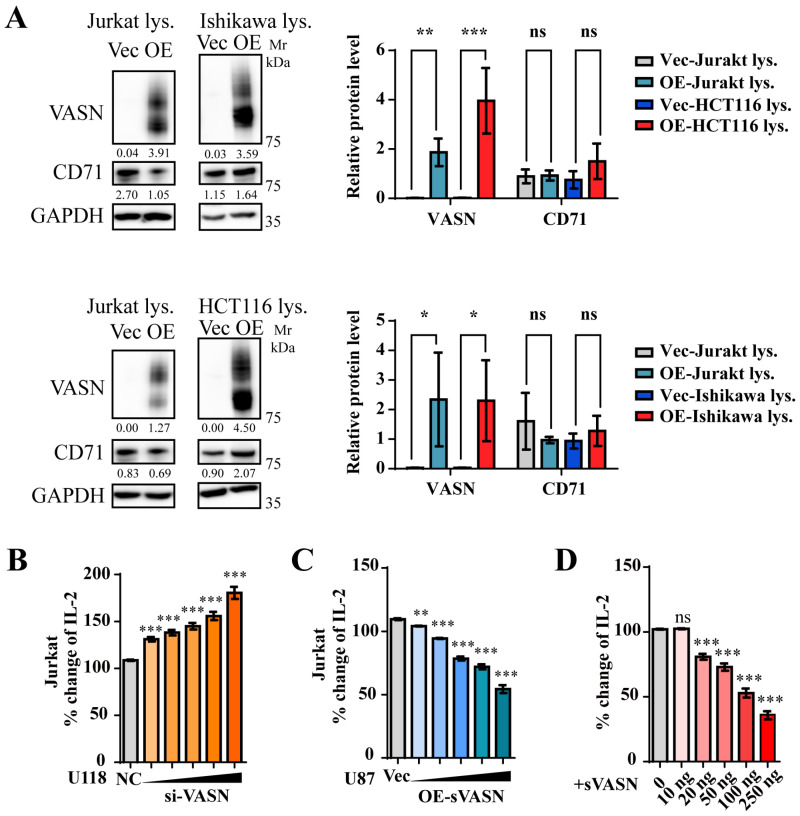
sVASN inhibited T cell activation. (**A**) Western blot analysis of the lysates of Ishikawa or HCT116 cells co-cultured with activated Jurkat cells. *n =* 3. (**B**) U118 cells were transfected with increasing doses of siRNA to knock down the VASN gene and were co-cultured with activated Jurkat cells. The fold change in the level of IL-2 was measured. (**C**) U87 cells were transfected with increasing doses of sVASN expression plasmids to overexpress the sVASN protein and were co-cultured with activated Jurkat cells. The fold change in the level of IL-2 was measured. (**D**) Activated Jurkat cells were treated with increasing doses of sVASN, and the fold change in IL-2 was measured. The Western blot bands were determined via ImageJ software. GAPDH was used as an internal control. All of the data are presented as the means ± SDs. All of the assays were conducted in triplicate. Statistical analysis in (**A**) was performed using two-way ANOVA followed by Tukey’s multiple comparisons test and in (**B**–**D**) using ordinary one-way ANOVA followed by Dunnett’s multiple comparisons test. * *p* < 0.05, ** *p* < 0.01, *** *p* < 0.001, ns not significant compared with the indicated groups.

We further asked whether and how sVASN affects T-cell function. We employed a co-culture system to examine the expression levels of VASN and CD71 in both cancer cells and T cells. The results revealed that the overexpression of sVASN in cancer cells significantly increased VASN protein levels in Jurkat cells (Figure 12A). To investigate the effects of sVASN secreted by cancer cells on T cells, the VASN level was manipulated in HepG2 cells for co-culture with activated Jurkat cells, as previously reported [36]. The IL-2 level increased when VASN was knocked down, whereas the IL-2 level decreased when VASN was overexpressed (Supplementary Figure S9A). Cancer cells with gradient descent (U118) or elevation (U87) levels of VASN were co-cultured with activated Jurkat cells to detect the inhibition of activated Jurkat cells. The results showed that the knockdown of VASN in U118 cells led to increased activation of Jurkat cells (Figure 12B), whereas the overexpression of sVASN in U87 cells resulted in attenuated activation of Jurkat cells (Figure 12C), indicating the immunosuppressive effects of sVASN within the tumor microenvironment. To clarify the role of sVASN, activated Jurkat cells were treated with exogenous sVASN directly. The expression of IL-2 decreased in a dose-dependent manner with increasing doses of sVASN (Figure 12D). We also examined the expression of CD26, a marker of T-cell activation, via flow cytometry. The results revealed that the expression of CD26 decreased as the concentration of sVASN increased (Supplementary Figure S9B).

**Figure 13:**
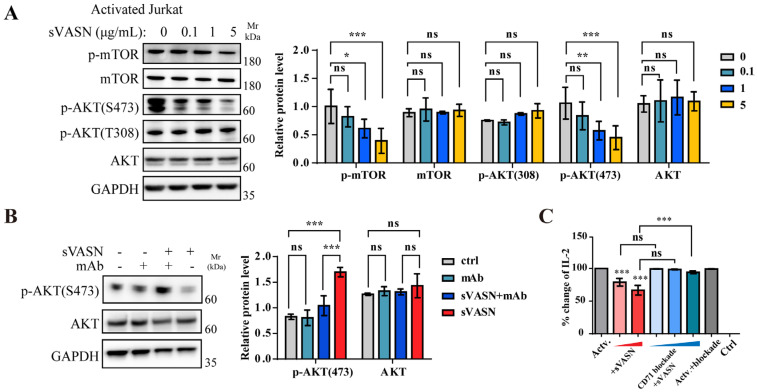
sVASN inhibited T cell activation through cell surface CD71. (**A**) Western blot analysis of the mTOR-AKT signaling pathway after sVASN treatment in activated Jurkat cells. *n =* 3. (**B**) Western blot analysis of the mTOR-AKT signaling pathway in activated Jurkat cells treated with sVASN with or without VASN-specific monoclonal antibody (mAb) counteraction. *n =* 3. (**C**) Activated Jurkat cells were treated with increasing doses of sVASN or CD71 blockade, and the fold change in the level of IL-2 was measured. Actv., activated Jurkat cells. GAPDH was used as an internal control. All of the data are presented as the means ± SDs. All of the assays were conducted in triplicate. Statistical analyses in (**A**,**B**) were performed using two-way ANOVA followed by Tukey’s multiple comparisons test and in (**C**) using ordinary one-way ANOVA followed by Tukey’s multiple comparisons test. * *p* < 0.05, ** *p* < 0.01, *** *p* < 0.001, ns not significant compared with the indicated groups.

Signaling pathway analysis in T cells revealed that in activated Jurkat cells, total mTOR and AKT levels remained stable, whereas phosphorylated mTOR (p-mTOR) and phosphorylated AKT (p-AKT, S473) sharply declined with increasing doses of sVASN (Figure 13A). We also employed the VASN monoclonal antibody to determine the specific role of sVASN in immunosuppression and demonstrated that the mAb reversed the attenuated p-AKT (S473) in activated Jurkat cells (Figure 13B). To determine whether the immunosuppressive effect of sVASN on T cells was mediated by CD71, a CD71-blocking peptide was used, as previously described. The immunosuppressive effect of sVASN on T cells was significantly impeded after CD71 was blocked, especially with high concentrations of blocking peptide (Figure 13C). Finally, we analyzed the immune checkpoint pairs in the co-culture system shown in Supplementary Figure S9A. T-cell-proliferation-related biological processes were enriched with VASN knockdown or sVASN overexpression (Supplementary Figure S10A,B), indicating that the immunosuppressive effect was dependent on sVASN. Our results provided evidence that sVASN suppressed T-cell activation through the mTOR-AKT signaling pathway via the cell surface CD71. Moreover, our study verified sVASN as a prospective anti-tumor target within the tumor microenvironment.

## 3. Discussion

The tumor microenvironment is a complex and dynamic ecosystem that surrounds and interacts with cancer cells. Targeting molecules within the TME has emerged as a promising approach for developing novel cancer therapeutic targets. One of the most well-known strategies is the use of checkpoint inhibitors. In the TME, immune checkpoint molecules such as programmed death-ligand 1 (PD-L1) on cancer cells and programmed cell death protein 1 (PD-1) on T cells play crucial roles in immunosuppression. Monoclonal antibodies that block the interaction between PD-L1 and PD-1, such as pembrolizumab and nivolumab, have shown remarkable success in treating various cancers [37]. In this study, we have conclusively demonstrated that sVASN plays a pivotal role in mediating intricate communication within the TME. Specifically, sVASN facilitates the malignant progression of tumor cells, augments angiogenesis in endothelial cells and impedes the activation of T cells, thereby exerting a multifaceted regulatory effect on tumor progression. More importantly, these effects of sVASN can be counteracted by either obstructing the endocytic pathway through blocking CD71 on the cell surface or by deploying a specific monoclonal antibody against sVASN. Given the comprehensive and multifaceted effects of sVASN within the TME, it has emerged as a promising target for TME-directed therapeutic interventions. Our findings not only enhance the understanding of the complex interplay within the TME but also hold significant potential for the development of novel anti-cancer strategies.

The “TGF-β paradox”, described as the unique characteristic of functionally switching roles from tumor suppressor to metastasis promoter, has attracted much attention in the field of cancer research [38,39]. The TGF-β paradox may be better elucidated by the involvement of sVASN, which has the ability to bind to TGF-β and disrupt the signaling cascade. In normal or near-physiological cellular contexts, TGF-β exerts a pivotal tumor-suppressive function by inhibiting cell proliferation and suppressing tumorigenesis through well-defined molecular mechanisms. However, during tumor progression, an excessive amount of sVASN antagonizes the TGF-β signaling pathway and is internalized into the cells through CD71 to regulate the malignant progression of tumors. As a consequence, the tumor-suppressive effects of TGF-β are abrogated, and it astonishingly transforms into a driver of tumor invasion and metastasis.

Our study demonstrated that the nuclear translocation of sVASN was dependent on p-STAT3 (Tyr705). Studies have shown that different phosphorylation site modifications of STAT3 have different effects on its nuclear translocation [23,40]. Nevertheless, our results proved that both the Tyr705- and the Ser727-phosphorylated residues of STAT3 were activated after sVASN treatment, while the nuclear translocation of STAT3 was attributed to Tyr705. However, the mechanisms underlying how VASN translocates into the nucleus and its functions require further investigation.

Communication between different cells occurs through diverse processes, including membrane-to-membrane contact, ligand–receptor recognition and the release of soluble mediators [41,42,43]. In this study, we found that the uptake of sVASN was dependent on the expression level of CD71, indicating that the endocytosis of sVASN via CD71 is universal across almost all cell types. The relationship between VASN and CD71 is more likely to involve a ligand–receptor interaction, such as that between PD-L1 and PD-1 in cancer cells and immune cells. This study identified CD71 as an endocytosis receptor of sVASN for cell communication in the TME. As a receptor of a certain ligand, sVASN can specifically bind to CD71, and there is a saturation state between the binding of sVASN to CD71. However, the binding of the ligand to the receptor enables the initiation of downstream signaling events, which mainly depend on the receptor, and the ligand serves as a trigger, which needs further investigation. Therefore, our study provides clues that CD71 may serve as a functional receptor of sVASN, which needs in-depth exploration in the future.

In summary, our study identified CD71 as a specific binding protein of sVASN that can be endocytosed into endothelial cells, epidermal cells, cancer cells or T cells. sVASN exerts multiple tumor-promoting effects, including the proliferation of cancer cells, angiogenesis of endothelial cells and immunosuppressive effects on T cells, to accelerate tumor progression. Our study demonstrated the promising potential of sVASN as a valuable drug target within the TME for cancer therapy.

## 4. Materials and Methods

### 4.1. Cell Lines and Cell Culture

Human cell lines were obtained from Procell (Wuhan, China) or the Cell Resource Center of Peking Union Medical College (Beijing, China). The cells were maintained in RPMI 1640 media, MEM or high-glucose DMEM (HyClone, Logan, UT, USA). All of the cells were cultured in media supplemented with 10% fetal bovine serum (Gibco, New York, NY, USA) and 1% penicillin/streptomycin (HyClone, Logan, UT, USA) under a 5% CO_2_ atmosphere.

### 4.2. Antibodies and Reagents

Human recombinant sVASN protein was customized by GenScript Inc. (Nanjing, China) and expressed in HEK293T expression hosts. The expression plasmids for CD71 (HG11020-CF) and STAT3 (HG10034-CF) were purchased from Sino Biological (Beijing, China). Customized plasmids were synthesized by Sangon Biotech (Shanghai, China). The CD71-blocking peptide was purchased from Abcam (ab101219, Cambridge, UK). Antibodies against VASN were purchased from Abcam (ab15686868) and used for Western blotting and immunofluorescence staining. Antibodies against CD71 were purchased from Abcam (ab214039, Cambridge, UK) for Western blotting and Invitrogen (13-6800, Carlsbad, CA, USA) for IF. Ferristatin II (Direct Black 38) was purchased from Med Chem Express (HY-D0256, MCE, Monmouth Junction, NJ, USA). Antibodies against Notch1 (3608), Lamin A/C (4777), YAP1/TAZ (8418), p-YAP (4911), AKT (9272), p-AKT-473 (4060), p-AKT-308 (9275), p-mTOR (2971), Stat3 (9139), p-Stat3-Ser727 (9134) and p-Stat3-Tyr705 (9145) were purchased from Cell Signaling Technology (CST, Danvers, MA, USA). Antibodies against Myc tag (D291), His tag (D291) and Flag tag (M185) were purchased from MBL Life Science (MBL, Nagoya, Aichi, Japan). Antibodies against GM130 (ab52649), Calnexin (ab22595), LAMP1 (ab24170), TGN46 (ab50595), c-MYC (ab32072), goat anti-Mouse IgG HL (Alexa Fluor 488, ab150113) antibody and goat anti-Rabbit IgG HL (Alexa Fluor 594, ab150080) were purchased from Abcam. Antibodies against Ang-1 (23302-1) and GAPDH (60004-1), β-actin (81115-1) and α-tubulin (11224-1) were purchased from Proteintech (Rosemont, IL, USA).

### 4.3. Cell Transfections

One day before transfection, cells were seeded in growth medium without antibiotics at a density of 60–70%. For gene knockdown, siRNA-sequence-targeting VASN as well as one negative control were obtained from Ribo Tech (genOFF st-h-VASN_002, stB0014993B-1-5, Guangzhou, China). Briefly, siRNA (100 nM) and negative control (NC) were each introduced into cells by using Lipofectamine RNAiMAX Transfection Reagent (Invitrogen, Carlsbad, CA, USA) according to the manufacturer’s instructions. For gene over-expression, pcDNA3.1-sVASN-His plasmids were introduced into cells by using Lipofectamine 3000.

### 4.4. Western Blot

Whole-cell lysates were obtained using M-PER™ Mammalian Protein Extraction Reagent containing 5–10 µL of protease inhibitor cocktail (Thermo Fisher Scientific, Waltham, MA, USA). Proteins (20–30 µg) were separated by 4–10% SimplePAGE Bis-Tris (Sangon Biotech, Shanghai, China) and transferred to PVDF membranes (0.2 µm, Millipore, MA, USA). The membrane was blocked with 5% non-fat dried milk in TBST at room temperature for 1 h before being incubated with antibodies in the blocking solution overnight at 4 °C followed by washing three times with TBST. The membrane was incubated with an HRP-conjugated secondary antibody at room temperature for 1 h and washed three times with TBST. Antigen–antibody complexes were detected using SuperSignal™ West Pico PLUS (Thermo Fisher Scientific, Waltham, MA, USA).

### 4.5. Co-Immunoprecipitation (Co-IP) Assays

HEK293T cells were transfected with the indicated plasmids for 48 h, after which the cell lysates were collected by centrifugation at 12,000× *g* for 10 min at 4 °C. The indicated antibodies and protein A/G magnetic beads (Bimake, Houston, TX, USA) were added to the supernatant, followed by incubation with gentle agitation at 4 °C overnight. The magnetic beads were then washed with cold lysis buffer 3–4 times, and the co-IP products were collected for Western blot analysis.

### 4.6. ELISA Assay

To analyze the binding kinetic characteristics of sVASN-CD71, CD71 or sVASN (10 µg/mL) were coated onto an ELISA plate at a volume of 100 µL/well with coating buffer (15 µM Na_2_CO_3_, 35 µM NaHCO_3_, pH 9.6) at 4 °C overnight. As part of the binding procedure, binding substrates at different concentrations (50, 40, 30, 20, 10, 5, 2.5 and 0 µg/mL) were added to each well and incubated at 37 °C for 2 h. For competition assay, the coating and binding substrates were used at a concentration of 10 µg/mL. The concentrations of the competitors (Tf or sVASN) ranged from 10 to 200 µg/mL. For saturation binding assay, CD71 was coated onto the cells at a concentration of 1 µg/mL. The concentrations of the binding substrate, sVASN, ranged from 3.125 to 200 µg/mL. The corresponding substrate antibodies were added and incubated at 37 °C for 1 h. The complex was incubated with the HRP-conjugated secondary antibody at room temperature for 1 h before washing three times with PBST at every step. Finally, TMB substrate solution was added and incubated for less than 20 min at 37 °C and protected from light before stop solution was added. The reaction was read spectrophotometrically at a wavelength of 570 nm. Binding, competition and saturation binding curves and scatter plots were generated using GraphPad Prism software (10.4.1). The equation for the one-site binding model [Y = Bmax × X/(K_d_ + X)] described the equilibrium binding of sVASN to CD71. Comparative graphs depict the Kd values (affinity constant) of sVASN and its receptor, CD71. The apparent Kd values of sVASN and CD71 were compared and plotted using GraphPad Prism software by nonlinear regression using a one-site binding model.

### 4.7. sVASN-CD71 Binding Kinetic Characterization Assay

The binding kinetics of the sVASN-CD71 complex were monitored via a Biacore T200 biosensor (Cytiva, Sunnyvale, CA, USA). Briefly, biotin-labeled CD71 (50 nM, 3.8 µg/mL, in 0.05% PBST, approximately 100 µL) was coated onto the streptavidin chip, which was then exposed to sVASN dissolved in 150 µL of PBST at concentrations of 15.625, 31.25, 62.5, 125, 250, 500 and 1000 nM. After association, the streptavidin chip was exchanged with a regeneration solution (10 mM Gly-HCl, pH 1.5).

### 4.8. Co-Culture of Cancer Cells with JURKAT CELLS

The desired number of Jurkat cells was activated with 200 ng/mL phorbol-12-myristate-13-acetate (PMA, HY-18739, MedChemExpress, MCE, Monmouth Junction, NJ, USA) and 300 ng/mL ionomycin (HY-13434, MedChemExpress, MCE, Monmouth Junction, NJ, USA) for 24 h. The concentration of IL-2 was measured via ELISA (RK00002, ABclonal, Wuhan, China). After cell transfection for 36 h, the cancer cells were washed with PBS, and activated Jurkat cells were added to the wells with fresh complete RPMI medium. Jurkat cells and cancer cells were co-cultured at a ratio of 2:1 or 4:1. After co-culture for another 24 h, the media were collected for ELISA analysis, and the cell lysates were prepared for Western blot analysis.

### 4.9. Nuclear Extraction and Fractionation

The cells were plated into 15 cm dishes and transfected (or co-transfected) with the indicated plasmids. The collected cells were washed twice with cold PBS, and nuclear and cytoplasmic lysates were separated via the Nuclear and Cytoplasmic Protein Extraction Kit (Beyotime, Shanghai, China) according to the manufacturer’s instructions.

### 4.10. Immunofluorescence Staining and Confocal Laser Scanning Microscopy (CLSM)

The cells were plated into 35 mm confocal dishes for 24 h, and the complete culture medium was replaced with culture media supplemented with 1% FBS. The cells were treated with (1) PBS, (2) sVASN (10 μg/mL) for 6 h, (3) CD71-blocking peptide (100 μg/mL) or (4) CD71-blocking peptide (100 μg/mL) for 6 h, followed by the addition of sVASN (10 μg/mL). The cells were washed with PBS 4–5 times and then fixed with methyl alcohol for 1 h at −20 °C before being blocked with QuickBlock™ Blocking Buffer for Immunol Staining (Beyotime, Shanghai, China) at 37 °C for 30 min. The cells were incubated with the primary antibody (1:50–200) overnight at 4 °C, followed by staining with the secondary antibodies for 2 h at room temperature. Finally, the cells were treated with DAPI solution (ready-to-use; Solarbio, Beijing, China) and covered with ProLong™ Glass Antifade Mountant (Thermo Fisher Scientific, Waltham, MA, USA). Fixed cells were visualized under a laser scanning confocal microscope (LSM Zeiss 900, Carl Zeiss, Germany) with 63 × oil objectives.

### 4.11. Cell Proliferation

Cell proliferation was assessed using the CCK-8 assay according to the manufacturer’s instructions (Dojindo, Kumamoto, Japan). Briefly, a total of 2000–5000 Ishikawa cells were counted and seeded into 96-well plates in a medium volume of 100 μL per well. The next day, fresh media without sera were used. Different concentrations of sVASN were added. The absorbance was measured with a spectrophotometer at a dual wavelength of 450/550 nm every day.

### 4.12. Cell Migration

The migratory ability of the cells was detected via an 8 μm pored, 6.5 mm polycarbonate transwell filter (Corning, New York, NY, USA). The cells were pretreated as follows: (1) PBS, (2) sVASN (10 μg/mL) for 6 h, (3) CD71-blocking peptide (100 μg/mL) or (4) CD71-blocking peptide (100 μg/mL) for 6 h, followed by the addition of sVASN (10 μg/mL). Pretreated cells were resuspended and counted (2–5 million cells) in serum-free culture medium and plated into the upper chamber. The lower chamber contained complete culture medium supplemented with 10% FBS (0.8 mL). After 18–36 h of incubation, the cells were fixed with paraformaldehyde and stained with 0.1% crystal violet (Beyotime, Shanghai, China) for 0.5 h. The fixed cells were visualized and counted via a microscope.

### 4.13. Wound Healing Assay

For the wound healing assay, a culture insert (µ-Dish 35 mm, ibidi, GmbH, Martinsried, Germany) was used for plating the cells. After the cells reached 80–90% confluency, the culture insert was carefully removed and treated with different conditions, as above, for another 12–24 h. Wound closure areas were calculated as follows: migration area (%) = (A0 − An)/A0 × 100, where A0 represents the initial wound area and An represents the remaining wound area at the measurement point.

### 4.14. Spheroid Formation

U251 cells were plated into 6-well ultralow attachment plates (Corning, New York, NY, USA) at a density of 5000 viable cells per well in spheroid medium at 37 °C in 5% CO_2_ under different conditions, as described above. The spheroid medium consisted of DMEM/F12 (Invitrogen, Carlsbad, CA, USA) supplemented with B27 serum-free supplement (1:50; Invitrogen), 20 ng/mL epidermal growth factor and basic fibroblast growth factor (R&D Systems, Minneapolis, MN, USA). The experiment was terminated after 10–14 days, and the spheroids were visualized and quantified.

### 4.15. Angiogenesis

The angiogenesis of sVASN was measured by using the Angiogenesis Assay Kit (In Vitro, Abcam, Cambridge, UK) according to the manufacturer’s instructions. Briefly, the extracellular matrix solution was added to 35 mm confocal dishes for 1 h at 37 °C to form a gel, and hCMEC/D3 cells were pretreated with different conditions, as above, for another 6–12 h. Tube formation was examined via fluorescence microscopy.

### 4.16. In Vivo Tumorigenesis

Animal experiments were performed with the approval of the Institutional Animal Care and Use Committee of the Laboratory Animal Center, Beijing Institute of Basic Medical Sciences (IACUC-DWZX-2023-P694). Female BALB/c nude mice (aged six weeks) were obtained from SPF Biotech (Beijing, China) and randomly divided into four groups (*n =* 5 per group). Four million Ishikawa cells were resuspended in 0.2 mL of PBS buffer and treated with different conditions, as above. The cells were then implanted into the posterior dorsal flank region of nude mice. The mice were maintained under standard conditions according to the institutional guidelines for animal care. Tumor length and width were measured every three days via a caliper. When the tumor volume reached approximately 1200 ± 30 mm^3^, the mice were sacrificed. The tumor volume was calculated via the following formula: tumor volume = (length × width^2^)/2.

### 4.17. Mass Spectrometry

Mass spectrometry (MS) was performed using ESI-QUAD-TOF to identify the binding protein of soluble VASN in the serum of AFP-negative hepatocellular carcinoma patients. The raw MS/MS data were converted into MGF format by Proteome Discoverer 1.2 (Thermo Fisher Scientific, Waltham, MA, USA). The exported MGF file data were analyzed by Mascot (Matrix Science, Boston, MA, USA) against the database containing all predicted human proteins. The peptide mass tolerance and the fragment mass tolerance were settled as ±0.2 Da. The results were described as follows: ions score is −10 × Log(P), where P is the probability that the observed match is a random event and individual ions scores >38 indicate identity or extensive homology (*p* < 0.05); protein scores were derived from ions scores as a non-probabilistic basis for ranking protein hits. The original data are filed in the Supporting Information.

### 4.18. Docking

The interaction docking simulation and molecular dynamics simulation for binding stability of sVASN to CD71 was conducted using Discovery Studio, ZDOCK. The crystal structure of CD71 was from PDB database (PDB Code:3KAS). The crystal structures of STAT3 and sVASN were predicted using the AlphaFold software V2. CD71 was set as the Receptor Protein and sVASN as the Ligand Protein. The parameters were as follows: Angular Step Size 15, ZRank Flase, RMSD Cutoff 6.0, Interface Cutoff 9.0 and Maximum Number of Clusters 60.

### 4.19. ChIP-Seq Analysis

The data were obtained from GEO database (GSM2278006, GSM2278007, GSM2278008, GSM2278009, GSM3754332, GSM3754333 and GSM3754334). Set indicated p-value and fold-enrichment thresholds to determine the DNA regions bound to STAT3 by using MACS2 software V2.2. The distribution of peaks across on the genome was visualized using IGV. We extracted the promoter regions of genes and analyzed the enrichment of peaks in the promoter regions to conduct GO (Gene Ontology) and KEGG (Kyoto Encyclopedia of Genes and Genomes) enrichment analysis.

### 4.20. Immunofluorescence Assay

Immunofluorescence (IF) staining assay was entrusted to Servicebio and performed as follows. Briefly, paraffin-embedded xenograft tumor tissue sections were de-paraffinized and rehydrated, followed by antigen retrieval. The primary antibodies were incubated overnight at 4 °C, followed by incubation with appropriate fluorescent-dye-labeled secondary antibodies at room temperature for 50 min. The nuclei were stained with 4, 6-diamidino-2-phenylindole (DAPI) (Servicebio, Wuhan, China), and the stained cells were imaged with a Nikon Fluorescence Microscope (DS-U3). For statistical analysis, the number of co-localization positive cells of the scans was visualized at 63× magnification in 6 random fields. Each violin extends from the lower to the upper quartile, where the horizontal line indicates the median value. The *y*-axis represents the percentage of co-localization cells, calculated as follows: positive rate of co-localization = number of co-localization cells/(total number of red positive cells + total number of green positive cells − total number of co-localization cells). All of the co-localization-positive areas were analyzed using Aipathwell (Servicebio, Wuhan, China). The co-localization of cells or the mean density in Figure S5 were analyzed as follows. The *y*-axis represents the percentage of co-localization cells, calculated as follows: positive rate of co-localization = number of co-localization cells/(total number of red positive cells + total number of green positive cells − total number of co-localization cells). The mean density = IOD/positive pixel area.

### 4.21. Statistical Analysis

All of the statistical analyses were performed by using GraphPad Prism 10.4.1 (GraphPad Software, San Diego, CA, USA). Two-group comparisons were made using an unpaired two-tailed Student’s t test. Multiple-group comparisons were conducted using one-way ANOVA or two-way ANOVA. The data are presented as the means ± standard deviations (SDs). Images of the immunoblots were analyzed via ImageJ (NIH, USA).

## 5. Conclusions

In conclusion, our study reveals a previously unrecognized mechanism by which sVASN acts as a tumor-promoting factor to accelerate tumor malignant progression through cell-surface CD71. By showing, for the first time, that exogenous sVASN could be internalized into cancerous, endothelial and T cells via CD71 on the cell surface, we provide novel insights into developing the drug target within the tumor microenvironment for cancer therapy. These findings not only enhance our understanding of the tumor-promoting role of sVASN to accelerate tumor malignant progression but also highlight the sVASN-CD71-STAT3 axis as a promising therapeutic strategy for future clinical applications.

## Data Availability

All data generated or analyzed during this study are indicated in this article. The datasets generated during and/or analyzed during the current study are available from the corresponding author upon reasonable request.

## Supplementary Materials

The following supporting information can be downloaded at: https://www.mdpi.com/article/10.3390/ijms26104913/s1.

## Abbreviations

The following abbreviations are used in this manuscript:

TME	Tumor microenvironment
MS	Mass spectrometry
aa	Amino acid
IP	Immunoprecipitation
NLS	Nuclear localization signal
GEO	Gene Expression Omnibus
PPI	Protein–protein interaction
