# Supplementary material for: CD71-Mediated Effects of Soluble Vasorin on Tumor Progression, Angiogenesis and Immunosuppression
Source: Int J Mol Sci. 2025 May 20;26(10):4913. doi: 10.3390/ijms26104913 (PMC12111917; doi:10.3390/ijms26104913)
Supplement: Supplementary file 1 [file ijms-26-04913-s001.zip › ijms-3623540-supplementary.pdf]

# Supplementary Information

## CD71−Mediated Effects of Soluble Vasorin on Tumor Progression, Angiogenesis and Immunosuppression

Yuechao Zhao †, Can Xiao †, Shaohua Li, Aixue Huang, Hui Li, Jie Dong, Qiaoping Qu, Xuemei Liu, Bo Gao * and Ningsheng Shao *

Department of Biochemistry and Molecular Biology, Beijing Institute of Basic Medical Sciences, Beijing 100850, China; zhaoyuechao@bmi.ac.cn (Y.Z.);

xiaocan@bmi.ac.cn (C.X.); shhli55@163.com (S.L.); huangaixue@bmi.ac.cn (A.H.); lihui1@bmi.ac.cn (H.L.); dongjie@bmi.ac.cn (J.D.); qvqiaoping@163.com (Q.Q.); liuxuemei@bmi.ac.cn (X.L.)

* Correspondence: gaobo1@bmi.ac.cn (B.G.); shaoningsheng@bmi.ac.cn (N.S.); Tel.: +86−13021060196 (B.G.); Tel: +86−13501111528 (N.S.); Fax: +86−1068213039 (N.S.)

† These authors contributed equally to this work.

**Keywords:** sVASN, CD71, tumor malignant, angiogenesis, immunosuppression

**This PDF file includes:**

Supplementary Figure S1 to S10

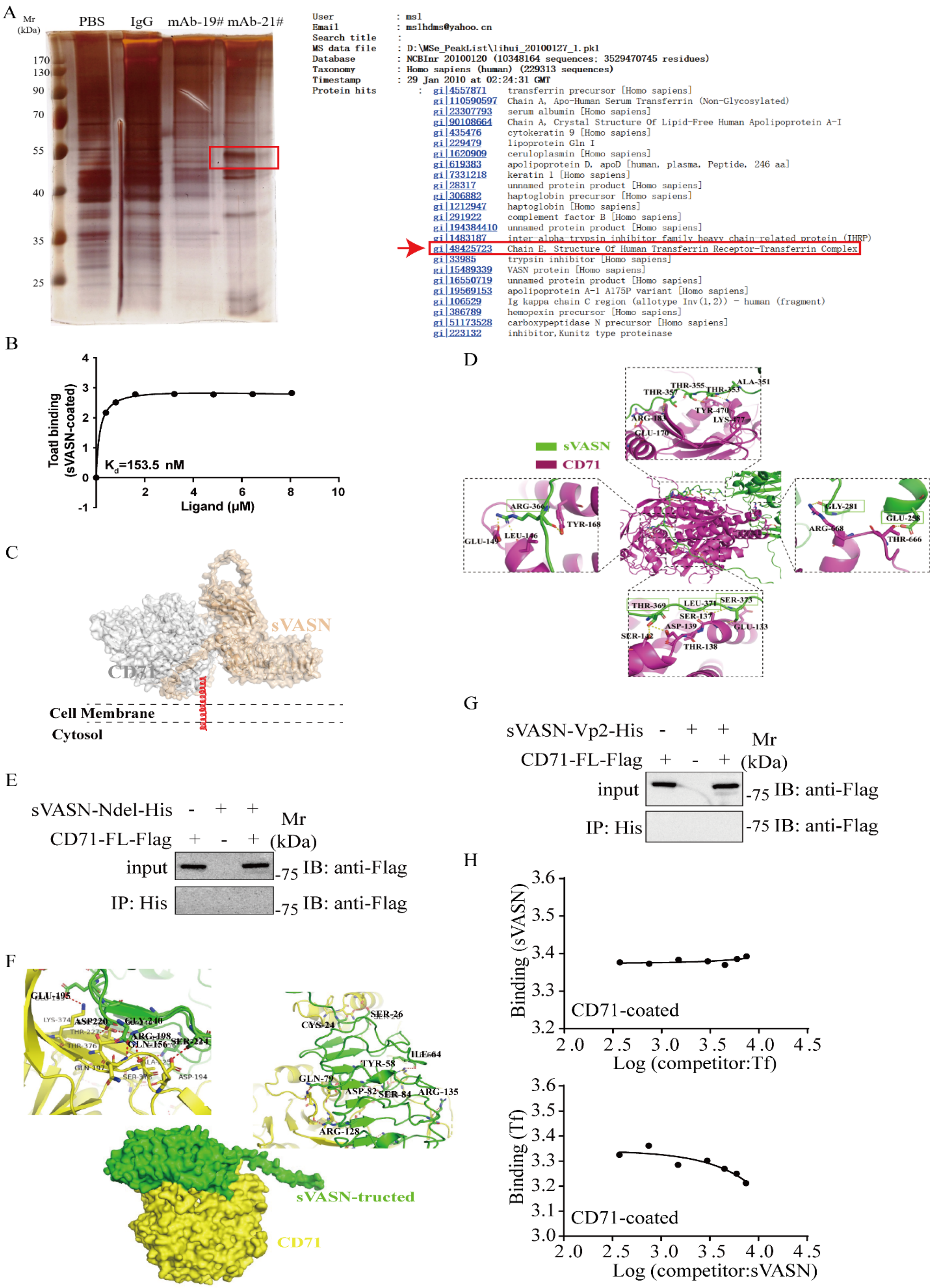
A
Mr (kDa)
PBS
IgG
mAb-19#
mAb-21#
170
130
90
70
55
40
35
25
User : msl
Email : mslhdms@yahoo.cn
Search title :
MS data file : D:\MSe_PeakList\lihui_20100127_1.pkl
Database : NCBInr 20100120 (10348164 sequences; 3529470745 residues)
Taxonomy : Homo sapiens (human) (229313 sequences)
Timestamp : 29 Jan 2010 at 02:24:31 GMT
Protein hits :
gi|4557871 transferrin precursor [Homo sapiens]
gi|110590597 Chain A, Apo-Human Serum Transferrin (Non-Glycosylated)
gi|23307793 serum albumin [Homo sapiens]
gi|90108664 Chain A, Crystal Structure Of Lipid-Free Human Apolipoprotein A-I
gi|435476 cytokeratin 9 [Homo sapiens]
gi|229479 lipoprotein Gln I
gi|1620909 ceruloplasmin [Homo sapiens]
gi|619383 apolipoprotein D, apoD [human, plasma, Peptide, 246 aa]
gi|7331218 keratin 1 [Homo sapiens]
gi|28317 unnamed protein product [Homo sapiens]
gi|306882 haptoglobin precursor [Homo sapiens]
gi|1212947 haptoglobin [Homo sapiens]
gi|291922 complement factor B [Homo sapiens]
gi|194384410 unnamed protein product [Homo sapiens]
gi|1483187 inter-alpha-trypsin inhibitor family heavy chain-related protein (IHRP)
gi|48425723 Chain E, Structure Of Human Transferrin Receptor-Transferrin Complex
gi|33985 trypsin inhibitor [Homo sapiens]
gi|15489339 VASN protein [Homo sapiens]
gi|16550719 unnamed protein product [Homo sapiens]
gi|19569153 apolipoprotein A-1 A175P variant [Homo sapiens]
gi|106529 Ig kappa chain C region (allotype Inv(1,2)) - human (fragment)
gi|386789 hemopexin precursor [Homo sapiens]
gi|51173528 carboxypeptidase N precursor [Homo sapiens]
gi|223132 inhibitor, Kunitz type proteinase
B
Toatl binding (sVASN-coated)
$K_d$=153.5 nM
Ligand (μM)
C
sVASN
CD71
Cell Membrane
Cytosol
D
sVASN
CD71
THR-357
THR-355
THR-353
ALA-351
ARG-183
TYR-470
LYS-477
GLU-170
ARG-366
TYR-168
LEU-146
GLU-149
GLY-281
GLU-258
ARG-668
THR-666
THR-369
LEU-371
SER-373
SER-137
ASP-139
GLU-133
SER-142
THR-138
E
sVASN-Ndel-His - + +
CD71-FL-Flag + - +
Mr (kDa)
input
IP: His
-75
IB: anti-Flag
F
GLU-195
ASP220
GLY-240
ARG-198
GLN-156
SER-224
CYS-24
SER-26
ILE-64
TYR-58
GLN-79
ASP-82
SER-84
ARG-135
ARG-128
sVASN-tructed
CD71
G
sVASN-Vp2-His - + +
CD71-FL-Flag + - +
Mr (kDa)
input
IP: His
-75
IB: anti-Flag
H
Binding (sVASN)
CD71-coated
Log (competitor:Tf)
Binding (Tf)
CD71-coated
Log (competitor:sVASN)

Supplementary Figure S1. CD71 was identified as a sVASN-binding protein. (A) IP-MS analysis of the binding protein of sVASN in the serum of AFP-negative hepatocellular carcinoma patients. The full data is available from the authors. (B) ELISA analysis of the binding of sVASN to CD71. sVASN was coated at the concentration of 10 μg/mL. The concentrations of CD71 were 50, 40, 30, 20, 10, 5, 2.5, 0 μg/mL, respectively. (C) Simulation docking of sVASN to cell surface CD71. The helix of CD71 was shown on the cell membrane. CD71 was shown in grey and sVASN was shown in brown yellow. (D) The predicted binding regions of sVASN to cell surface CD71. CD71 was shown in magenta and sVASN was shown in green. The detailed interaction residues of sVASN were labeled with green box. (E) Co-IP analysis of the binding of sVASN-Ndel to full-length CD71. sVASN-Ndel, the C-terminal of sVASN, 85-240 aa of VASN with His tag in the C-terminal. (F) Simulation docking of sVASN-truncted structure (1-240 aa) to cell surface CD71. CD71 was shown in yellow and sVASN-truncated was shown in green. The detailed interaction residues of sVASN were labeled. (G) Co-IP analysis of the binding of sVASN-Vp2 to full-length CD71. sVASN-Vp2, 120-240 aa of VASN with His tag in the C-terminal. (H) ELISA competition binding assay. The coating and binding substrate was at the same concentration of 10 μg/mL. The concentration of competitors (Tf or sVASN) ranged from 200 μg/mL to 10 μg/mL. The assays were conducted in triplicate.

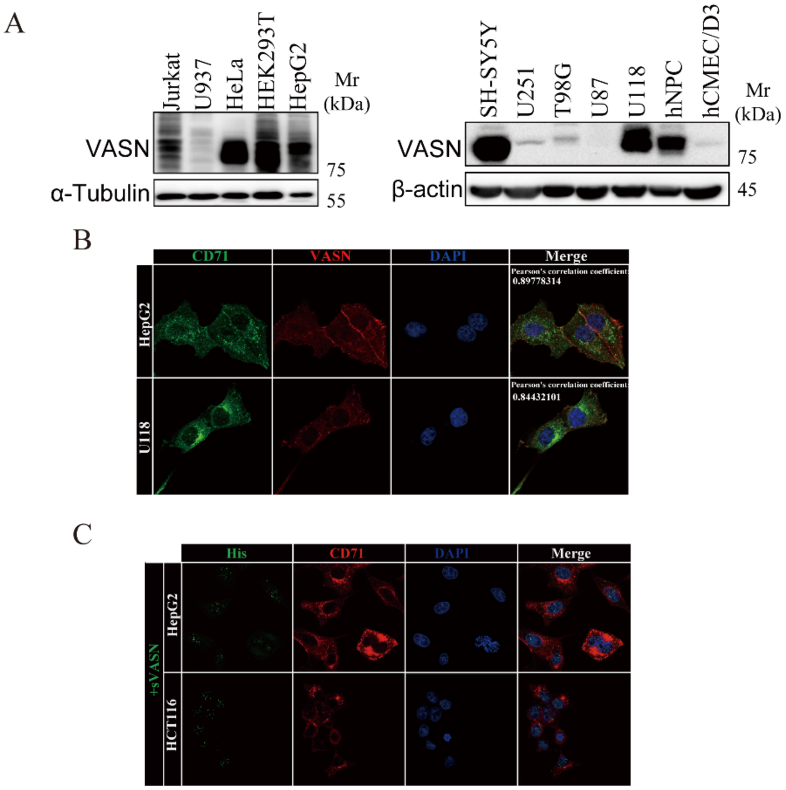


Supplementary Figure S2. Exogenous sVASN could be internalized into different types of cells. (A) Western blot analysis the constitutive protein levels of VASN in different types of cells. α-tubulin or β-actin was used as an internal control. (B) The colocalization of VASN and cell-surface CD71 in fixed cells, which was captured under a LSCM using a 63×/1.4NA objective. The Pearson correlation coefficient was determined via ImageJ software. (C) The internalization of exogenous sVASN into different types of cells visualized under a LSCM using a 63×/1.4NA objective.

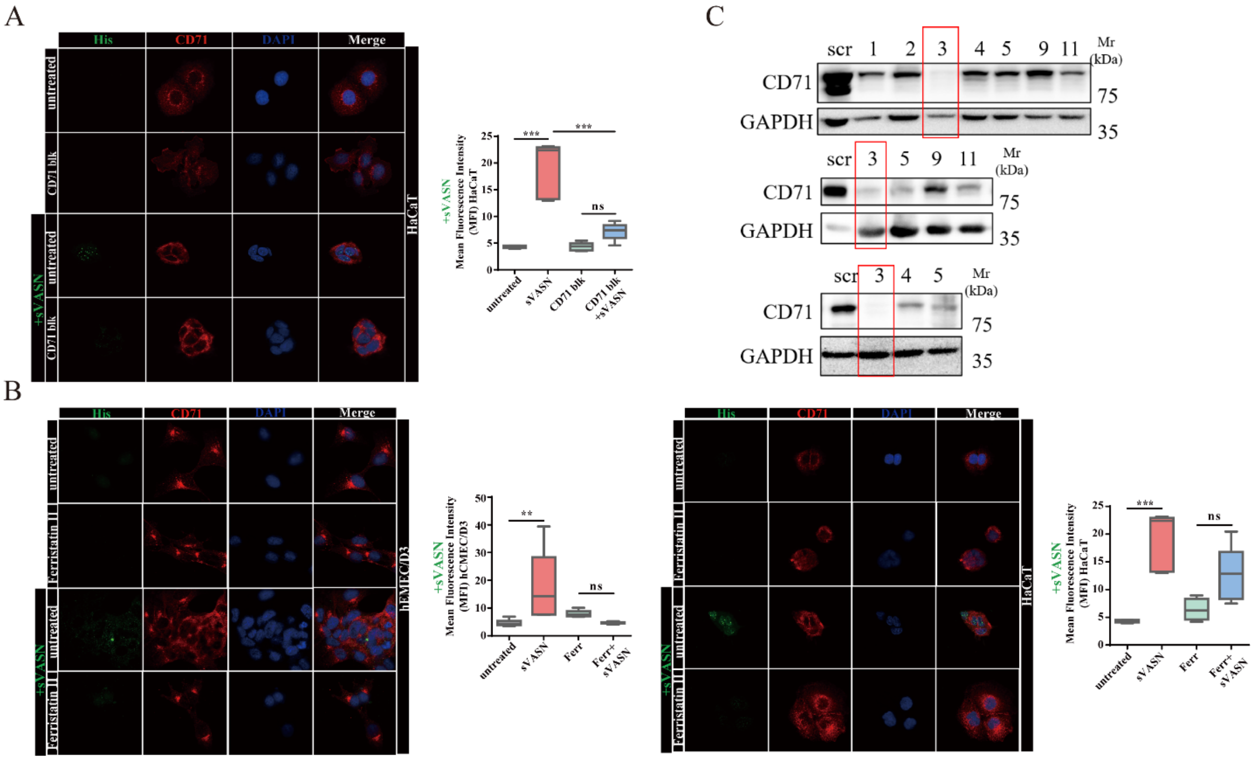


Supplementary Figure S3. Exogenous sVASN could be internalized into different types of cells through cell surface CD71. (A) The internalization of exogenous sVASN in HcCaT cells after blocking cell-surface CD71 visualized under a LSCM using a 63×/1.4NA objective. (B) The internalization of exogenous sVASN after inhibiting the expression of CD71 in hCMEC/D3 (left) or HaCaT (right) cells visualized under a LSCM using a 63×/1.4NA objective. (C) Western blot analysis the CD71 protein levels of the monoclonal cells using the CRISPR/Cas9 method. All data are mean ± SD. All assays were conducted in triplicate. Statistical analysis in (A) and (B) were performed with ordinary one-way ANOVA followed by Tukey's multiple comparisons test. *p < 0.05, **p < 0.01, ***p < 0.001, ns not significant compared with the indicated groups.

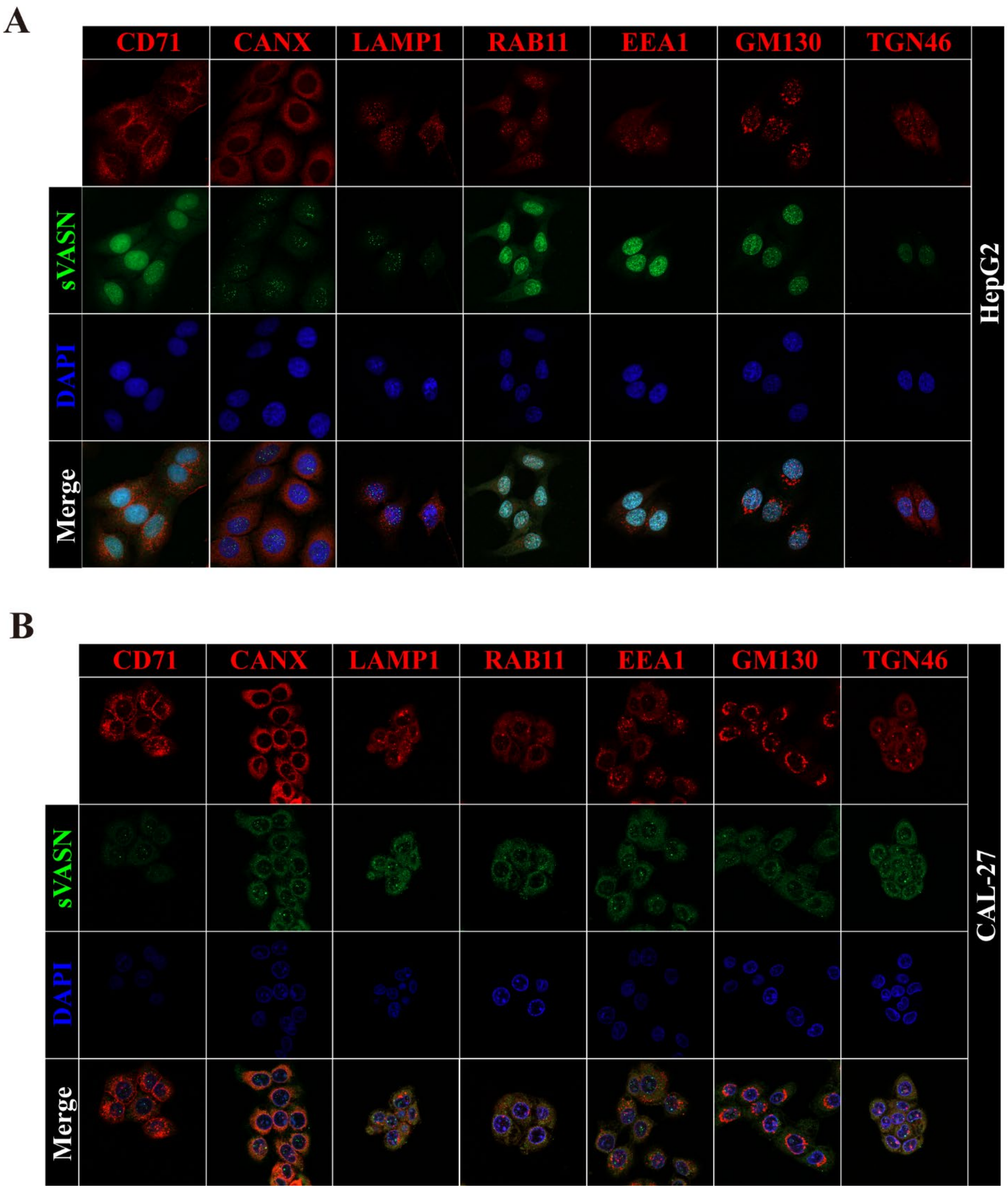


Supplementary Figure S4. Colocalization of sVASN and different recycling endosomes in HepG2 cells and CAL-27 cells visualized via LSCM. (A-B) Endoplasmic reticulum (CANX), late endosome (LAMP1), recycling endosome (RAB11), early endosome (EEA1) and Golgi (GM130 and TGN46). The assays were conducted in triplicate.

A

B

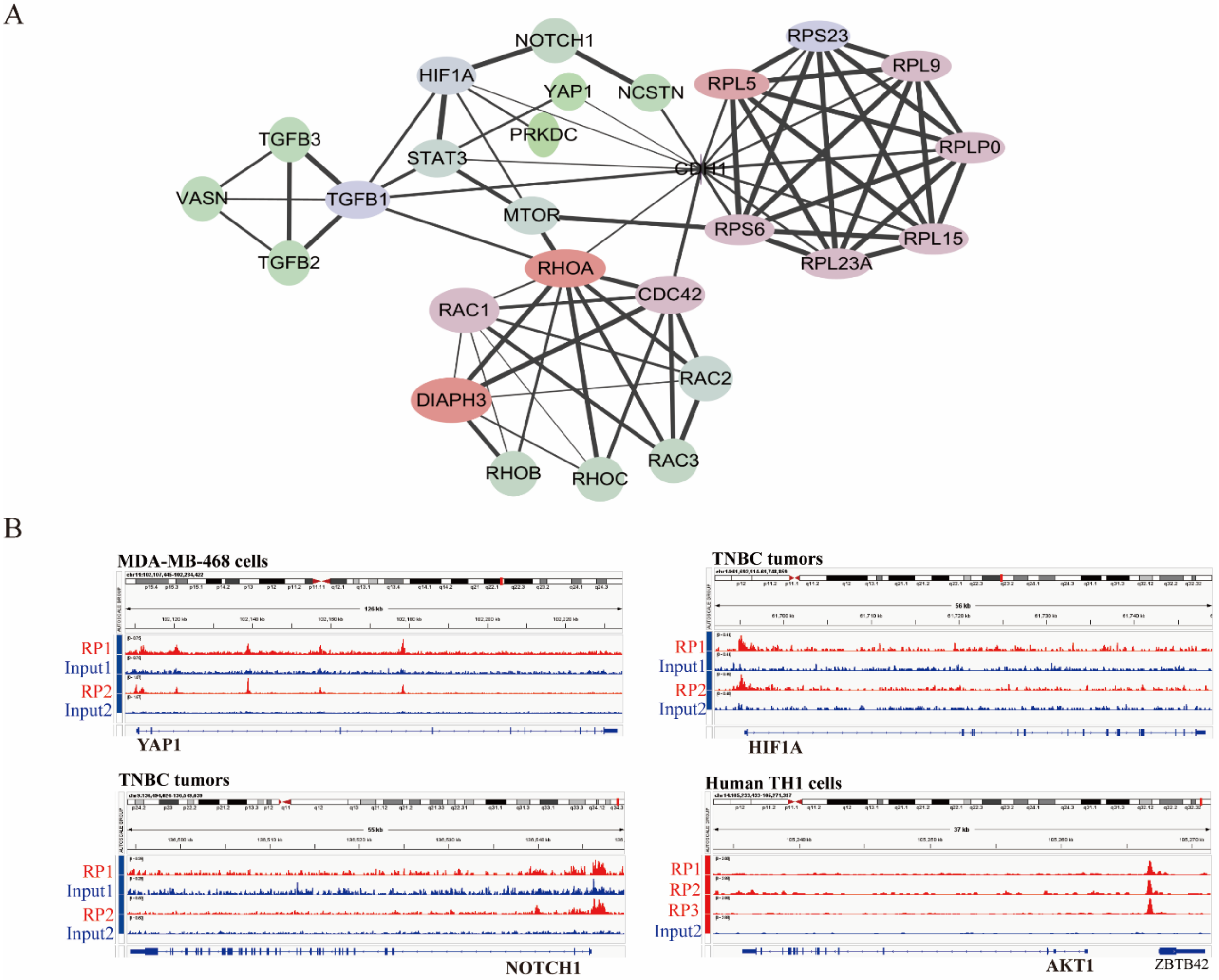


Supplementary Figure S5. Endocytosed sVASN enhanced the nuclear translocation of STAT3. (A) The PPI networks of VASN with related proteins obtained from STRING and BioGrid database. (B) An overview of the produced ChIP-seq data illustrated by IGV of the indicated gene promoter.

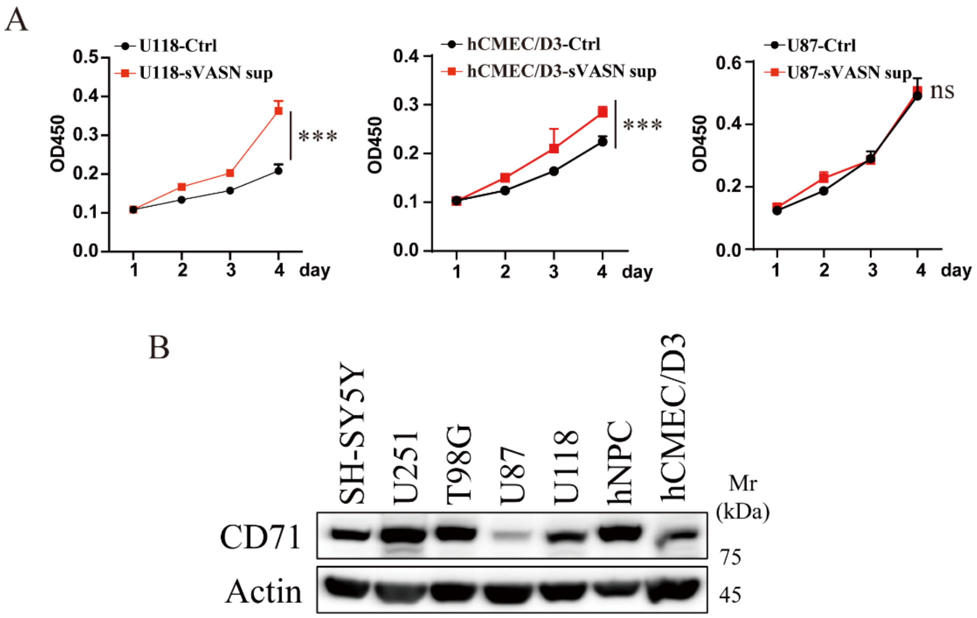


Supplementary Figure S6. Exogenous sVASN was conducive to the proliferation of cancer cells through cell surface CD71. (A) HepG2 cell supernatant promoted cell proliferation of U118 and hCMEC/D3 cells but had little effect on U87 cells. (B) Western blot analysis of the constitutive protein levels of CD71 in different cell lines. Bands of Western blot were determined by Image J software. β-actin was used as an internal control. Statistical analysis in (A) was performed with two-way ANOVA test. *$p < 0.05$, **$p < 0.01$, ***$p < 0.001$, ns not significant compared with the indicated groups.

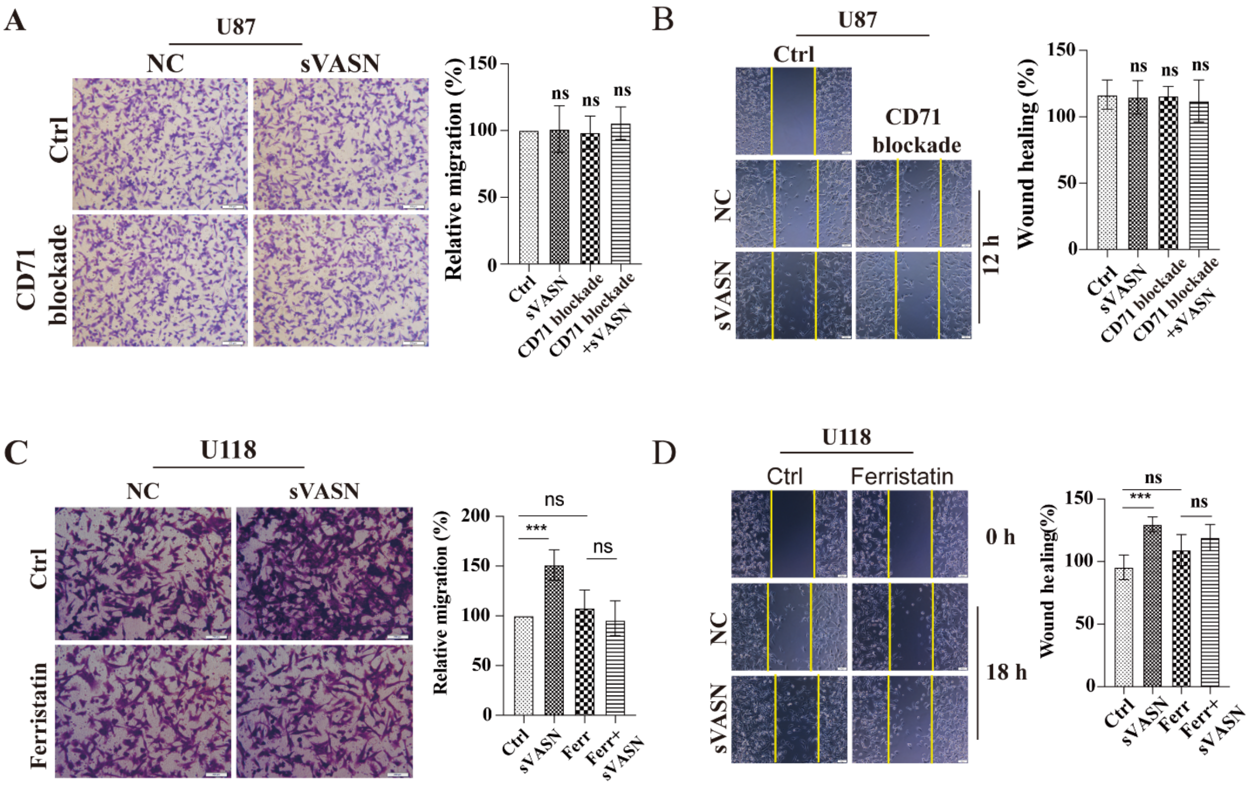


Supplementary Figure S7. Exogenous sVASN was conducive to the migration of cancer cells through cell surface CD71. (A-B) Migration (A) and wound healing (B) assay of U87 cells treated with exogenous sVASN, CD71 blocking peptide, CD71 blocking peptide followed by sVASN, or blank, respectively. Representative images (left) and statistics (right) were shown. (C-D) Migration (C) and wound healing (D) assay of U118 cells treated with exogenous sVASN, Ferristatin II, Ferristatin II followed by sVASN, or blank, respectively. Representative images (left) and statistics (right) were shown. Statistical analysis in (A-B) and (C-D) were performed with ordinary one-way ANOVA followed by Tukey's multiple comparisons test. *$p < 0.05$, **$p < 0.01$, ***$p < 0.001$, ns not significant compared with the indicated groups.

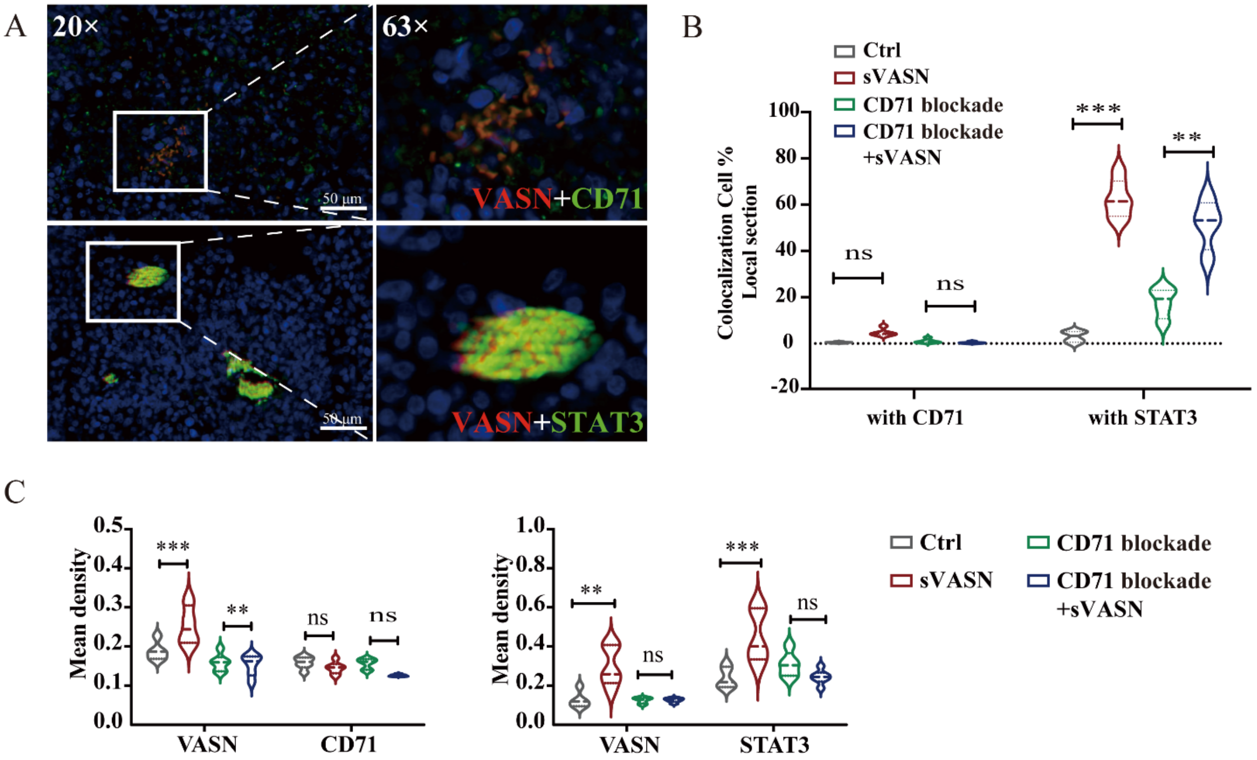


Supplementary Figure S8. the immunofluorescence staining (IF) of VASN with CD71 or STAT3 *in vivo*. (A) The local section scans of the immunofluorescence staining of VASN (red) with CD71 (green) or STAT3 (green), using the tumor bulk obtained from the tumorigenesis assay. (B) Violin plot comparison of the percentage of co-localization cells of VASN with CD71, or with STAT3 in the six random views of the section scans, respectively. (C) Violin plot comparison of the mean density of indicated proteins in the staining scans of VASN with CD71 or STAT3, respectively. Statistical analysis in (B) was performed with two-way ANOVA followed by Tukey's multiple comparisons test, (C) with two-way ANOVA followed by Sidak's (left) or Tukey's (right) multiple comparisons test. *$p < 0.05$, **$p < 0.01$, ***$p < 0.001$, ns not significant compared with the indicated groups.

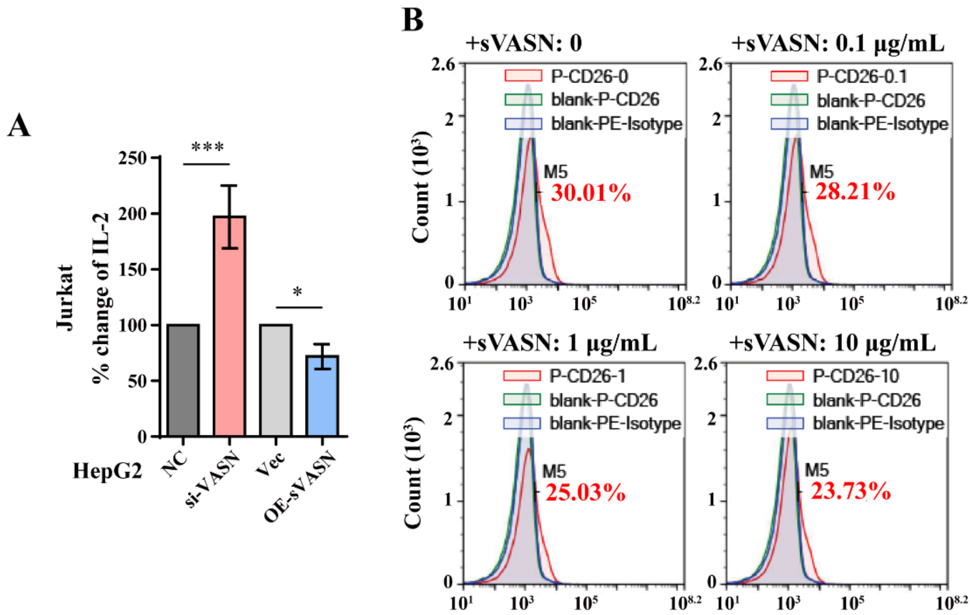


Supplementary Figure S9. sVASN inhibited T cell activation. (A) HepG2 cells were transfected with specific si-RNA to knock-down VASN gene or sVASN expression plasmids to overexpress sVASN protein and co-cultured with activated Jurkat cells. The fold change of IL-2 was measured. (B) FACS analysis of the CD26 levels on the surface of activated Jurkat cells with increasing doses of sVASN treatment. Red line, CD26 staining; blue line, isotype control staining; green line, untreated control staining. Statistical analysis in (A) was performed with ordinary one-way ANOVA followed by Tukey's multiple comparisons test. *p < 0.05, **p < 0.01, ***p < 0.001, ns not significant compared with the indicated groups.

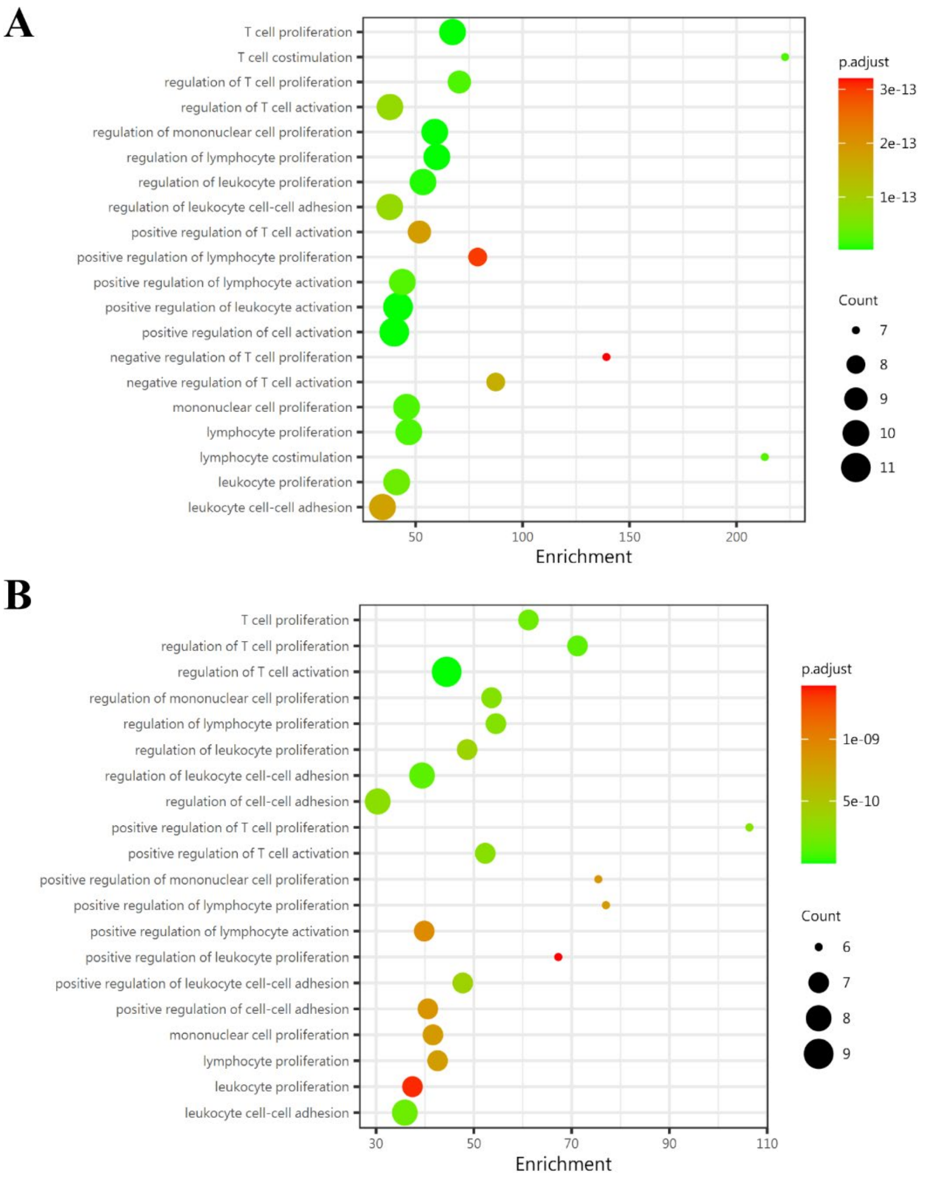


Supplementary Figure S10. sVASN inhibited T cell activation through cell surface CD71. (A-B) The supernatant of the co-culture system in (A) was collected for Human Immune Checkpoint Array (AAH-ICM-1, RayBiotech) assay. The biological process (BP) was enriched in activated Jurkat cells co-cultured with VASN knock-down HepG2 cells (A), or sVASN over-expression HepG2 cells (B), respectively.
